# Alloimmune Responses of Humanized Mice to Human Pluripotent Stem Cell Therapeutics

**DOI:** 10.1016/j.celrep.2017.08.003

**Published:** 2017-08-22

**Authors:** Nigel G. Kooreman, Patricia E. de Almeida, Jonathan P. Stack, Raman V. Nelakanti, Sebastian Diecke, Ning-Yi Shao, Rutger-Jan Swijnenburg, Veronica Sanchez-Freire, Elena Matsa, Chun Liu, Andrew J. Connolly, Jaap F. Hamming, Paul H.A. Quax, Michael A. Brehm, Dale L. Greiner, Leonard D. Shultz, Joseph C. Wu

**Affiliations:** 1 Stanford Cardiovascular Institute, Stanford University School of Medicine, Stanford, California, USA; 2 Department of Medicine, Stanford University School of Medicine, Stanford, California, USA; 3 Institute for Stem Cell Biology and Regenerative Medicine, Stanford University School of Medicine, Stanford, California, USA; 4 Department of Comparative Medicine, Stanford University School of Medicine, Stanford, California, USA; 5 Department of Pathology, Stanford University School of Medicine, Stanford, California, USA; 6 Department of Surgery, Leiden University Medical Center, Leiden, the Netherlands; 7 Diabetes Center of Excellence, Department of Molecular Medicine, University of Massachusetts Medical School, Worcester, MA, USA; 8 The Jackson Laboratory, Bar Harbor, ME, USA

**Keywords:** pluripotent stem cells, stem cell therapeutics, allograft, immunogenicity, humanized mice, T cell exhaustion, wasting disease

## Abstract

There is growing interest in using embryonic stem cell (ESC) and induced pluripotent stem cell (iPSC) derivatives for tissue regeneration. However, an increased understanding of human immune responses to stem cell-derived allografts is necessary for maintaining long-term graft persistence. To model this alloimmunity, humanized mice engrafted with human hematopoietic and immune cells could prove to be useful. In this study, an in-depth analysis of graft-infiltrating human lymphocytes and splenocytes revealed that humanized mice incompletely model human immune responses toward allogeneic stem cells and their derivatives. Furthermore, using an “allogenized” mouse model, we show the feasibility of reconstituting immunodeficient mice with a functional mouse immune system and describe a key role of innate immune cells in the rejection of mouse stem cell allografts.

## Highlights

• Innate immunity is crucial in rejection of minor HA mismatched grafts
• Stem cell alloimmune responses modeled with an “allogenized mouse”
• Humanized mice are unable to fully model immune responses to stem cell allografts
• Splenocytes and graft-infiltrating lymphocytes display an exhausted phenotype

## Graphical Abstract

**Figure:**
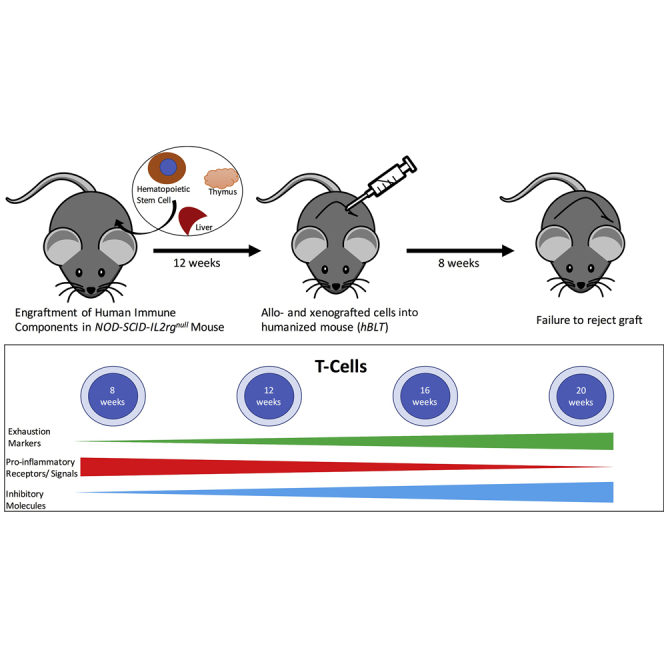

## Teaser

Kooreman et al. use various types of humanized mice for the modeling of pluripotent stem cell alloimmunity. They report the development of a wasting disease-like syndrome within these mice over time, limiting their functionality, and provide ways to address this by using an immune reconstituted “allogenized” mouse model.

## Introduction

Since the first isolation of human embryonic stem cells (hESCs) (Thomson et al., 1998) and creation of induced pluripotent stem cells (iPSCs) (Takahashi et al., 2007, Takahashi and Yamanaka, 2006), the field of regenerative medicine has been investigating the therapeutic potential of these cells for cardiac diseases (Nguyen et al., 2016), neurological diseases (Barberi et al., 2003), hepatic failure (Soto-Gutiérrez et al., 2006), diabetes (Pagliuca et al., 2014), and macular degeneration (Homma et al., 2013). Human clinical trials in immune-privileged areas, such as the eye for macular degeneration, are ongoing for ESC derivatives (Schwartz et al., 2012) or iPSC derivatives (Mandai et al., 2017). However, the immunological responses toward these derivatives in less immune-privileged sites are still poorly understood (de Almeida et al., 2013). Recently, advances have been made in tolerizing mice to accept human ESC- and iPSC-derived progenitor grafts for long-term monitoring of graft behavior (Lui et al., 2014). However, to date, it is still not clear how the human immune system would respond to allogeneic human ESC or iPSC grafts. This question would need to be answered before pluripotent stem cell (PSC) therapy, including both ESCs and iPSCs, could be widely implemented in clinical practice.

To model human immune responses, researchers have been studying immunodeficient mice engrafted with human immune cells and their progenitors, such as peripheral blood mononuclear cells (PBMCs) and hematopoietic stem cells (HSCs). The first description of these “humanized mouse models” dates back to 1983, when it was reported that the *Prkdc*^*scid*^ (severe combined immunodeficiency, *scid)* mutation in CB17 mice caused B and T cell deficiency (Bosma et al., 1983) and suggested that CB17-*scid* mice would be permissive for human HSC and PBMC engraftment. However, because of the high levels of host natural killer (NK) cell activity and the spontaneous generation of mouse B and T cells, this model supported only low levels of human HSC engraftment (Greiner et al., 1998). With the expression of human-like SIRPA in the non-obese diabetic (NOD)-*scid* strain, the levels of murine NK cells decreased (Shultz et al., 1995, Takenaka et al., 2007), resulting in heightened engraftment of human PBMCs (Hesselton et al., 1995). However, residual activity of NK cells as well as other innate immune system functions interfered with human HSC engraftment. Moreover, NOD-*scid* mice developed spontaneous thymic lymphomas, resulting in a shortened lifespan. It was not until the NOD-*scid* mouse strain with the interleukin-2 receptor gamma chain (*IL2rg*)-targeted mutation (NOD.Cg-*Prkdc*^*scid*^ *IL2rg*^*tm1Wj1*^/Sz, NOD scid gamma [NSG]) and related NOD/shi-scid/γc ^null^ (NOG) strain mice were repopulated with human HSCs (*scid*-repopulating cell, *hSRC* mouse), that superior human hematopoietic and immune cell engraftment was achieved (Ishikawa et al., 2005, Ito et al., 2002, Shultz et al., 2005).

Despite enhanced engraftment of human HSCs in immunodeficient *IL2rg*^*null*^ mice, a robust human T cell-mediated immune response could not be established (Traggiai et al., 2004). The relatively weak T cell response was hypothesized to be due to the lack of human leukocyte antigen (HLA) on the murine thymus that is necessary for the positive selection of human T cells. To address this, a new model was created by subcapsular renal implantation of human liver and thymus fragments as well as intravenous injection of autologous (human liver-derived) HSCs in sublethally irradiated immunodeficient mice and was termed the human bone marrow, liver, and thymus (*hBLT)* model (Lan et al., 2006, Melkus et al., 2006). The superior engraftment of human immune cells combined with positive selection of T cells in the autologous human thymus has made this the preferred model for studying human immune responses to infection (Brehm et al., 2014).

An emerging field where humanized mice could prove to be useful is the study of human immune responses to allogeneic PSC transplants to assess the efficacy and safety of PSCs and guide effective immunosuppressive therapies. Here we describe the use of *hSRC* and *hBLT* humanized NSG mice to model the human immune response to allogeneic hESCs and their derivatives. We track allograft survival over time using bioluminescence imaging (BLI). In addition, we provide large transcriptome data as well as single-cell immunological analysis of human graft-infiltrating T cells and splenocytes isolated from humanized mice. Furthermore, using a similar implantation of mouse liver, thymus, and bone marrow, we developed an “allogenized” mouse model as a surrogate to assess allogeneic immunological responses to murine PSC allografts in vivo and ex vivo.

## Results

### Human Immune-Engrafted NSG Mice Are Unable to Completely Reject Allogeneic hESCs

We used both the *hSRC* (NSG mice engrafted with HLA-A2^neg^ HSCs) and *hBLT* (NSG mice engrafted with HLA-A2^neg^ HSCs and fetal tissue) to model the allogeneic human immune responses to HLA-mismatched (HLA-A2^pos^) hESCs. The hESCs were stably transduced with a reporter construct containing the ubiquitin promoter driving firefly luciferase (Luc) and EGFP. Allogeneic HLA-A2^pos^ hESCs (1 × 10^5^) were implanted either subcutaneously (s.c.) or intramuscularly (i.m.) into *hSRC* mice. The hESC survival in these mice, as well as in control non-engrafted NSG and immunocompetent C57BL/6 mice, was longitudinally monitored in vivo using BLI. Both the *hSRC* and non-engrafted NSG mice were unable to completely reject allogeneic hESCs implanted at either injection site, whereas the immunocompetent C57BL/6 mice completely rejected the hESC grafts within 2 weeks (Figures S1A, S1C, S1D, and S1F).

To investigate whether low expression of major histocompatibility complex class I (MHC class I) in hESCs played a role in the failure of *hSRC* mice to reject these cells, hESCs were treated with interferon gamma (IFN-γ) for 24 hr prior to implantation into *hSRC* mice to increase expression of MHC class I and cell immunogenicity (Drukker et al., 2002). MHC class I, encompassing HLA A, B, and C in humans, encodes the main molecular targets of allograft rejection as well as MHC-associated incompatibilities between donor and recipient. It is also responsible for almost all acute rejection. Indeed, upregulation of MHC class I, as well as multiple other co-stimulatory molecules, was seen in hESCs upon stimulation with IFN-γ (Figure S2). However, even the IFN-γ-stimulated hESCs were not rejected by *hSRC* mice (Figures S1B, S1C, S1E, and S1F). To address the possibility that the inability to reject these hESCs may be due to the hESCs modulating the immune response locally and enforcing tolerance, we transplanted *hSCR* mice with murine ESCs (mESCs), which should normally be rejected by human immune cells. However, these humanized mice were unable to reject murine cells as well (Figures S1G and S1H).

Having provided evidence for the inability of the *hSRC* model to mount strong PSC-directed immune responses, we moved to the *hBLT* model. *hBLT* mice support robust human cell engraftment of mouse lymphoid tissues and development of functional human T lymphocytes (Lan et al., 2006, Melkus et al., 2006). Indeed, total human leukocyte engraftment in *hBLT* mice in peripheral blood 12 weeks after humanization showed superior engraftment of B cells and CD4^+^ T cells compared with the *hSRC* model (Figures S3A and S3B).

**Figure 1:**
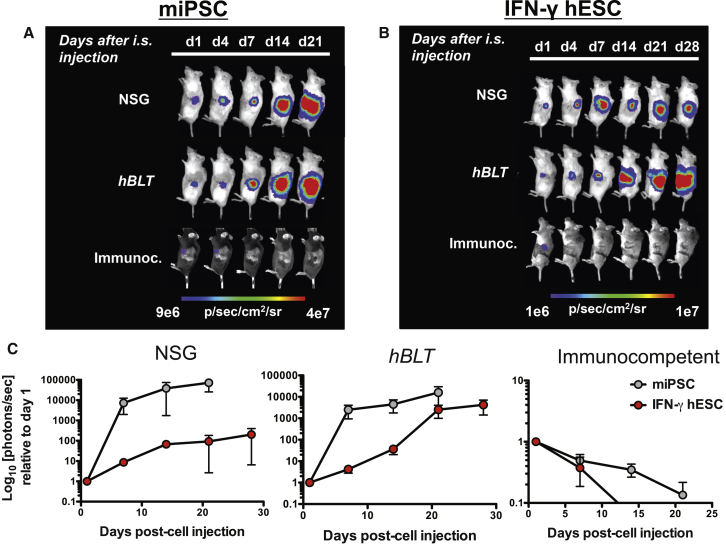
*hBLT* Mice Fail to Completely Reject Mouse iPSC Grafts and Allogeneic Human ESC Grafts (A and B) Representative BLI from one mouse per group, demonstrating the proliferation of miPSCs (A), and IFN-γ-hESCs (B) after i.s. injection in NSG (n = 3) and *hBLT* mice (n = 5) and rejection of these cells in the immunocompetent (C57BL/6) recipients (n = 4). (C) Quantification of the BLI data normalized to the maximum radiance on day 1, showing intensification of the signal over time for transplanted miPSCs as well as IFN-γ-hESCs in NSG and *hBLT* mice, respectively, resulting in teratoma formation (left and center). In contrast, complete immune rejection of miPSCs and IFN-γ-hESCs occurred in immunocompetent C57BL/6 mice by 21 and 14 days, respectively (right). BLI data are represented as mean ± SEM of two independent experiments.

We next tested the ability of *hBLT* mice to reject human and mouse PSCs. Similar to the *hSRC* model, *hBLT* mice were transplanted with mouse iPSC (miPSC) grafts, generated from fibroblasts of a transgenic FVB mouse ubiquitously expressing EGFP and Luc (de Almeida et al., 2014; Figure 1A), or with human IFN-γ-stimulated hESC allografts (Figure 1B) by intra-splenic (i.s.) injection. The survival of the grafts was again monitored using BLI, with the signal normalized to the maximum radiance on day 1 of transplantation (Figure 1C). Despite superior engraftment of human immune cell subsets in *hBLT* mice compared with *hSRC* mice, the murine and human grafts were not rejected and underwent proliferative growth that resulted in large teratoma formation by week 4 and week 3, respectively (Figure S3C).

### hBLT Mice Showed a Correlation between Pro-inflammatory Graft Infiltration and Graft Loss but Were Unable to Fully Reject Differentiated hESC-EC Grafts

**Figure 2:**
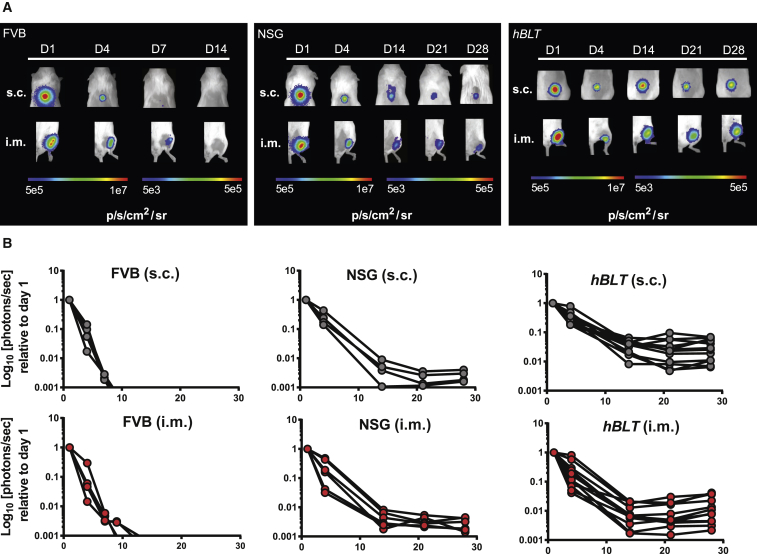
*hBLT* Mice Are Unable to Reject hESC-EC Grafts (A) Representative BLI from one animal per group over the course of 4 weeks, showing persistence of hESC-ECs after s.c. and i.m. injection in NSG (center, n = 5) and *hBLT* (right, n = 12) mice, whereas the immunocompetent FVB mice (left, n = 4) completely rejected the grafts by day 14. (B) Quantification of the BLI data normalized to the maximum radiance on day 1, showing initial non-immune-mediated graft loss followed by graft persistence in the subcutaneous (top) and gastrocnemius muscle (bottom) injection sites in NSG and *hBLT* mice and complete rejection by the FVB mice.

Next, we derived mature endothelial cells from the labeled hESC line (hESC-ECs) to upregulate MHC class I molecules on ESCs, which has been described during differentiation of hESCs (Drukker et al., 2002). Upregulation of MHC class I molecules as well as several other co-stimulatory molecules was confirmed by differentiating hESCs using the embryoid body method (Figure S2). hESC-ECs (1 × 10^5^) were then either transplanted s.c. in the back or i.m. in the gastrocnemius muscle of *hBLT* mice at 20 weeks after humanization, and graft survival was measured over time using BLI (Figure 2A). An initial decline in signal, representative of non-immune-mediated cell stress and loss during injections, was followed by stabilization of the signal and persistence of the graft for the duration of the study. Within the grafts, there appeared to be minimal immune cell infiltration (Figures S3D and S3E). The signal kinetics of hESC-ECs in *hBLT* mice were very similar or, in some cases, even improved compared with those in non-engrafted NSG mice, indicating that the reconstituted immune system might even favor graft persistence. In contrast, the signal in immunocompetent FVB mice returned to baseline within 2 weeks, indicating complete rejection of the hESC-EC grafts (Figure 2B). To be sure that the limited immune response was not the result of possible immune-evasive properties of hESC-ECs, we performed an additional experiment by transplanting labeled somatic human umbilical vein endothelial cells (HUVECs) into 16-week-old *hBLT* mice. Similar to the hESC-EC experiment, *hBLT* mice were unable to fully reject the HUVECs with graft persistence over a course of 3 weeks, whereas immunocompetent FVB mice rejected the cells within a week after transplantation (Figure S4**)**.

**Figure 3:**
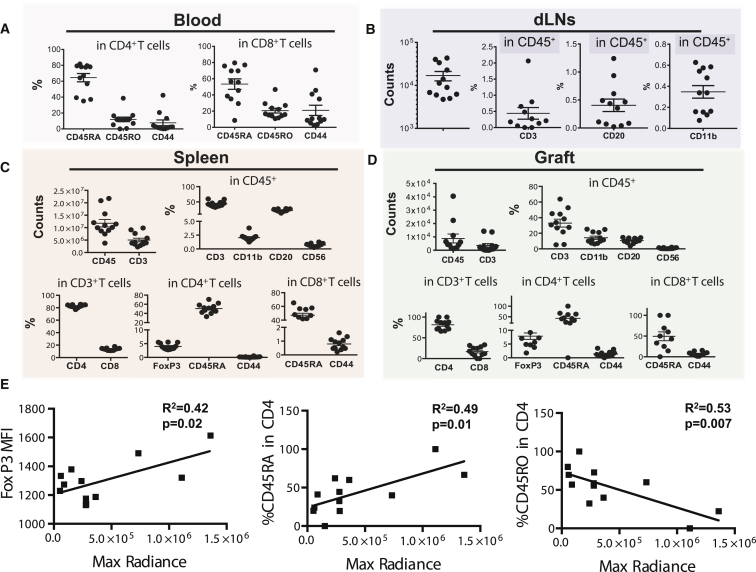
*hBLT* Mice Reveal Systemic Immune Activation, with a Strong Correlation between Small Numbers of Human Graft-Infiltrating Cells and Rejection of Allogeneic hESC-ECs (A and B) FACS analysis of (A) blood shows an active (CD45RO^+^; CD44^+^) systemic cytotoxic T cell (CD8^+^) response but (B) little population of T cells as well as B cells and macrophages in the inguinal dLNs. (C) Analysis of the spleen reveals larger populations of B cells (CD20^+^) and T cells (CD3^+^), but subpopulations of the latter show relatively high numbers of naive (CD45RA) and few effector memory (CD44^+^) T cells. (D) Limited numbers of graft-infiltrating lymphocytes consist mainly of naive T cells with few effector memory cells. (E) The decrease in BLI signal (Max Radiance) in the graft was significantly correlated with a lower number of regulatory T cells (FoxP3, left) and naive T helper cells (CD45RA, center) and an increase in activated T helper cells (CD45RO, right). Data are displayed as percentage (%) or number of cells (counts) within the parent population, e.g. in CD45/CD3/CD4/CD8 cells. Data are represented as mean ± SEM from one experiment (n = 12).

It is important to note that, over the course of the 4-week experiments, the condition of the *hBLT* mice deteriorated, with decreased body weight, fur loss, and lessened physical activity. For three *hBLT* mice, this necessitated euthanization. Uncertain about the cause of this increased morbidity and mortality, we analyzed the blood, spleen, draining inguinal lymph nodes (dLNs), and PSC grafts for human immune cell populations in the remaining 12 *hBLT* mice at 4 weeks (Table S1). Blood analyses of total CD4^+^ helper T cells showed the presence of 64.5% ± 5.2% naive (CD45RA^+^) T cells, 11.9% ± 2.8% activated memory helper T cells (CD45RO^+^), and 7.6% ± 3.6% effector memory (CD44^+^) helper T cells (Figure 3A, left). For CD8^+^ cytotoxic T cells, the levels of naive cells decreased to 53.4% ± 6.4%, and they gained a more activated memory function with CD45RO^+^ T cell levels at 20.6% ± 2.9% and cytotoxic effector memory cells at 21.0% ± 6.3% (Figure 3A, right). Only a very small percentage of human CD3^+^ cells (0.4% ± 0.1%) was found in the dLNs, indicating that active human cells found in the systemic circulation were unable to traffic or localize appropriately to the dLNs (Figure 3B). In addition, the dLNs contained very few human B cells or antigen-presenting cells. Analysis of the spleens showed substantial engraftment of human B and T lymphocytes but few monocytes or NK cells (Figure 3C). The subpopulations of CD3^+^ lymphocytes consisted mainly of helper T cells and few cytotoxic T cells that were largely naive, with very few effector memory cells observed. Within the grafts, the human immune cells mainly retained a naive phenotype, with small percentages of effector memory CD4^+^ (1.3% ± 0.3%) and CD8^+^ (7.0% ± 1.3%) cells (Figure 3D). However, there was a strong correlation between graft loss, as indicated by the relationship between the decay in BLI signal, a reduction in the number of regulatory FoxP3^+^ T cells and naive T cells (Figure 3E, left and center), and an increase in the amount of activated immune cells in the graft (Figure 3E, right). Again, the presence of inactive and immunosuppressive immune cells in the graft environment might favor graft persistence, whereas activated immune cells facilitated graft loss.

Taken together, our data suggest that these mice had an activated systemic immune system in the blood, but these active immune cells were not seen in similar quantities in the spleen, dLNs, or graft. Nevertheless, the limited numbers of activated graft-infiltrating T cells correlated strongly with graft loss. This revealed some functionality of the immune system in *hBLT* mice but also showed that they are incapable of mounting an effective alloimmune response to completely reject hESC-ECs.

### In-depth Analyses of Immune Cells from hESC-EC-Engrafted hBLT Mice Show a “Non-stimulated” T Cell Phenotype

Immune cells isolated from spleens, dLNs, and Matrigel plugs from hESC-EC-engrafted mice were used for mixed lymphocyte reactions to assess the effectiveness of the different lymphoid structures in mounting an immune response. First, immune cells isolated from the blood and spleen of *hBLT* mice were stimulated with phorbol ester (phorbol myristate acetate [PMA])/ionomycin, and their activation was compared with positive control samples of HiCK-1 cells and PBMCs isolated from human whole blood (Figure S5A). Even though some pro-inflammatory responses were seen in the splenocytes of *hBLT* mice, this was reduced compared with the positive control samples. Second, immune cells isolated from the Matrigel plugs and dLNs were incubated with hESC-EC lysate or HUVEC lysate to compare specific and non-specific immune responses (Figure S5B*)*. In this experiment, neither hESC-EC nor HUVEC lysate exposure resulted in immune cell activation. Moreover, non-antigen stimulation by PMA/ionomycin also did not result in T cell activation. We next analyzed the gene expression profile of the small population of activated graft-infiltrating lymphocytes. As negative and positive controls for T cell activation, PBMCs from a healthy human donor remained unstimulated or were stimulated in vitro with PMA/ionomycin for 72 hr. After 72 hr, the PBMCs were harvested and, together with the *hBLT* lymphocytes, stained for murine and human CD45 markers and human CD3, CD4, CD8, CD45RA, and CD45RO. Human lymphocytes with an activated memory surface marker profile (CD4^+^CD45RO^+^; CD8^+^CD45RO^+^) were isolated using fluorescence-activated cell sorting (FACS), and their gene expression was analyzed with the Fluidigm single-cell PCR platform using a panel of 92 genes known to be involved with human Th1, Th2, and Th3 immune responses (Table S2).

**Figure 4:**
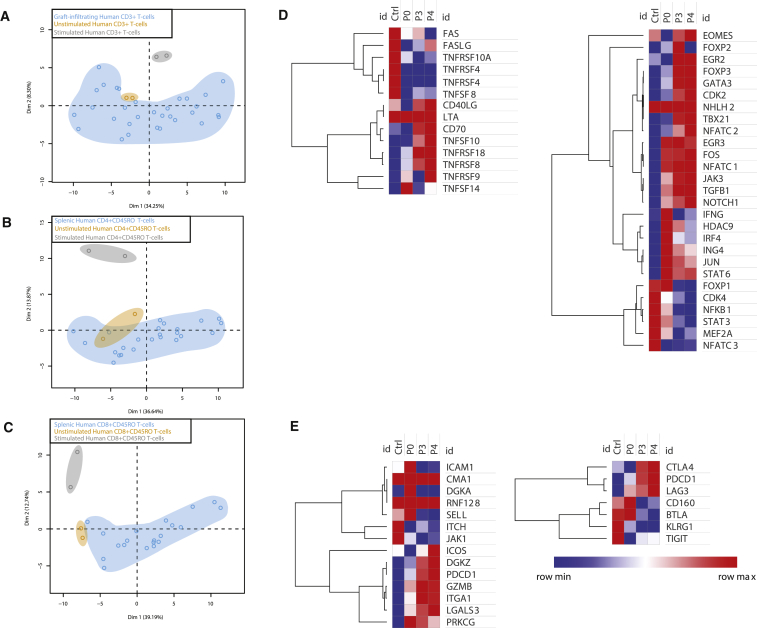
Single-Cell PCR Analysis and RNA-Seq of *hBLT* Graft-Infiltrating T Lymphocytes, dLNs, and Splenocytes Reveals the Unstimulated Phenotype (A) Graft-infiltrating lymphocyte (CD3^+^, blue) group with unstimulated human PBMCs (yellow) and wide separation of PMA/ionomycin-stimulated human PBMCs (gray). (B and C) A similar trend was seen for activated helper T cells (CD4^+^CD45RO, B) and activated cytotoxic T cells (CD8^+^CD45RO, C) isolated from the spleens of *hBLT* mice. (D) Human immune cells isolated from *hBLT* mice show an impaired immune profile compared with the human control samples. Human T cells were isolated from *hBLT* blood at week 8 (p0), week 16 (p3), and week 20 (p4) (n = 5 mice per time point) after humanization, and the RNA expression profile was compared with T cells isolated from healthy human blood samples. At p0, the immune profile of *hBLT* T cells is most similar to control T cells, despite some marked increases in pro-inflammatory cytokine profiles at that time point. In contrast, other genes involved in T cell inhibition (*FOXP3*, *TGFB1*) are hardly expressed at baseline, but their expression increases over time during the aging of the mice. Interestingly, at p3, the ESC-derived ECs and HUVECs were engrafted, but this did not result in changes in the immune profile at p4. (E) Human T cells isolated from *hBLT* mice develop T cell anergy and senescence upon aging. At p0, the T cell anergy gene profile of human T cells is similar to human control T cells. However, upon aging of the *hBLT* mice, the anergic profile becomes increasingly present (left). A similar result was seen for the inhibitory molecules (right). Also, the engraftment of allogeneic cells at p3 did not result in an alteration from an inhibitory to a pro-inflammatory immune profile.

Principal-component analysis (PCA) of the fold changes of all 92 genes revealed that *hBLT* CD3^+^ lymphocytes isolated from the grafts grouped with the unstimulated control human T lymphocytes (Figure 4A). Activated human helper T cells (CD4^+^CD45RO^+^) and cytotoxic T cells (CD8^+^CD45RO^+^) isolated from *hBLT* spleens showed a similar grouping to their unstimulated counterparts from the healthy control (Figures 4B and 4C). These activated memory graft-infiltrating lymphocytes and splenocytes had an “unstimulated” phenotype upon allogeneic antigen stimulus. However, the strong correlation between graft loss and the presence of CD45RO^+^ lymphocytes seems to indicate that the graft-infiltrating lymphocytes were properly stimulated to target the grafts earlier in the response but that the immune response had subsided. Therefore, we analyzed gene expression profiles in *hBLT* splenocytes associated with T cell anergy and exhaustion. T cell exhaustion is described in cases of chronic infection and continuous exposure to foreign antigens and is associated with the upregulation of genes such as *CTLA4*, *LAG3*, and *TIM3* (Wherry, 2011). Human activated memory helper T cells (CD4^+^CD45RO^+^) and activated memory cytotoxic T cells (CD8^+^CD45RO^+^) isolated from the spleen showed higher expression levels of these genes compared with unstimulated and stimulated PBMCs from the healthy human control (Figure S5C), providing initial signs for exhaustion of the human lymphocytes. A full-scale immune response cannot be mounted by these CD4^+^CD45RO^+^ and CD8^+^CD45RO^+^ lymphocytes, potentially explaining the inability of the *hBLT* model to fully reject allogeneic hESC grafts.

To assess where in the development of the *hBLT* model the failure in graft rejection occurs, we next analyzed the immune cells of *hBLT* mice (n = 5) at different time points during the humanization process. Blood samples from five *hBLT* mice were drawn at week 8 (p0), 16 (p3), and 20 (p4) after humanization, and human T cells were isolated for RNA sequencing (RNA-seq). The immune profile was then compared with T cells from a healthy control human blood sample (ctrl). The immune profile of human immune cells within the *hBLT* model at week 8 post-humanization reveals an immune profile similar to the human control sample, but, by weeks 16 and 20, this immune profile starts to diverge and shows significant upregulation of immune profiles associated with T cell anergy and upregulation of inhibitory molecules (Figures 4D and 4E).

### Allogenized Mice Have a More Organized Lymphoid Architecture Than Humanized Mice and Can Reject Xenogeneic and Allogeneic iPSCs

Our data so far have demonstrated the superior engraftment of human immune cells in the *hBLT* model, but the model appears to have a limited ability to mount a robust full-scale allogeneic immune response to human PSCs over time. To determine whether this is “model-dependent,” we next created allogenized mouse models by reconstituting NSG mice with allogeneic C57BL/6 mouse bone marrow (*aBM* mice) as well as mouse bone marrow, liver, and thymus (*aBLT* mice). Donor immune cells, as well as fetal liver and thymus transplanted in the abdomen, remained viable for up to one year in these models (Figure S6A).

Engraftment of donor immune cells in allogenized NSG mice was assessed by flow cytometry 12 weeks post-HSC transplantation. Donor cells were identified based on the CD45 allelic disparity between donor C57BL/6 (CD45.2) and recipient NSG (CD45.1) mice. Both the *aBM* and the *aBLT* allogenized mouse models had high levels of donor immune cell engraftment. The percentages of total leukocytes, T cells, B cells, and NK cells in peripheral blood were similar between the two models and did not differ from wild-type C57BL/6 mice (Figure S6B).

**Figure 5:**
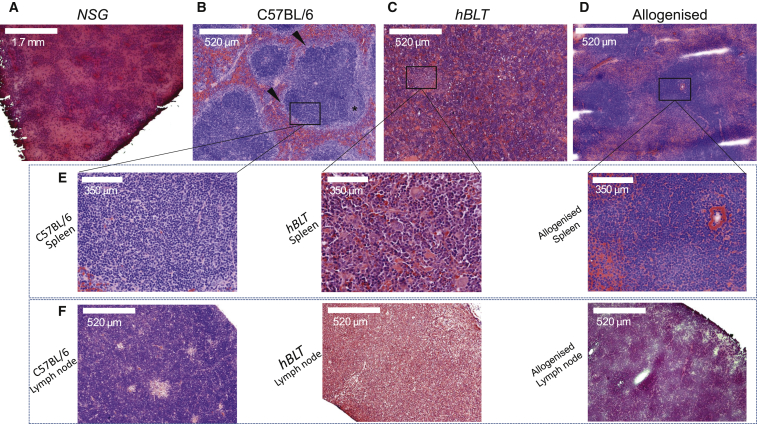
Allogenized Mice Have a More Organized Lymphoid Architecture that Is Similar to Wild-Type Mice (A) Overview of splenic tissue in NSG mice with poorly developed splenic architecture (H&E, 2× magnification). (B) Normal splenic architecture in a wild-type C57BL/6 mouse with a periarteriolar T cell area, B cell area (asterisk), and marginal zone (arrowheads) (H&E, 10× magnification). (C) *hBLT* mice show a high degree of hematopoiesis in the spleen but little periarteriolar T cell migration and an overall disorganized splenic architecture (H&E, 10× magnification). (D) Allogenized mice have an architecture more similar to the wild-type C57BL/6 mouse, with periarteriolar lymphoid sheets. However, apparent germinal center B cell areas as well as marginal zones are missing, giving the periarteriolar margins an irregular shape (H&E, 10× magnification). (E) Higher-magnification images (40×) of sites of interest in (B)–(D). (F) Overview of inguinal dLNs, showing high levels of lymphocyte infiltration in wild-type C57BL/6 (left) and allogenized mice (right) compared with the *hBLT* mouse model (center) (H&E, 40× magnification).

We examined the lymphoid structures in *aBLT* mice and compared these with the lymphoid structures in the *hBLT* mice, wild-type C57BL/6 mice, and unmanipulated negative control NSG mice (Figure 5A). H&E analysis of the spleens of *hBLT* mice revealed marked hematopoiesis but minimal periarteriolar lymphocyte migration (Figures 5C and 5E, center). Interestingly, this splenic architecture does mature over time with the development of a marginal zone by week 24 after humanization (Figure S7). In dLNs, human lymphocytes were found in minimal quantities at week 12 compared with the wild-type mouse (Figure 5F, left and center). In contrast, the spleen of the allogenized mouse at week 12 after humanization had a very similar architecture as the wild-type C57BL/6 mouse (Figures 5B and 5E), with periarteriolar lymphoid sheaths but without apparent germinal center B cell areas. In addition, the lymphoid areas of allogenized mouse spleens had irregular margins, consistent with missing marginal zone-type cell development or paucity of the perilymphoid macrophage-rich collections that are part of the specialized open and closed circulation (Figures 5D and 5E, right). Analysis of the dLNs in the allogenized mouse revealed high levels of lymphocytes, indicating migration of the lymphocytes to peripheral lymphoid organs (Figure 5F, right).

**Figure 6:**
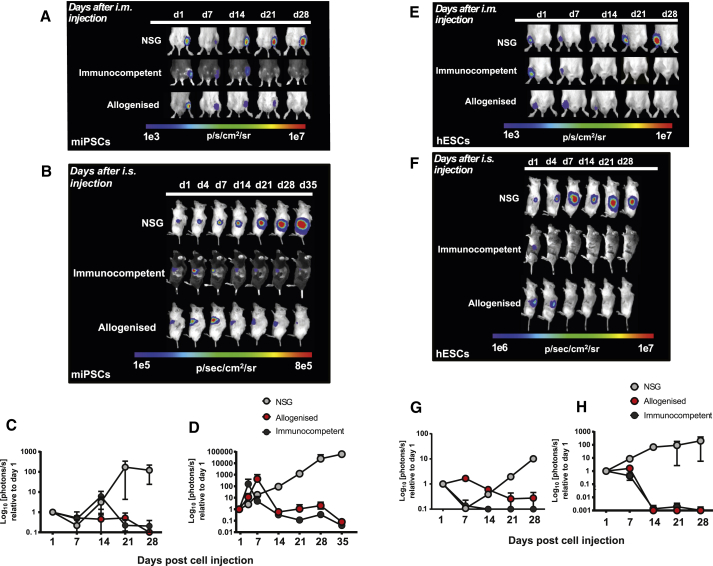
Allogenized Mice Show Robust Rejection of Allogeneic miPSC Grafts and Xenogeneic Human IFN-γ-Stimulated ESC Grafts (A) Robust but somewhat delayed rejection of miPSC allografts injected i.m. (B) miPSC allografts injected i.s. were rejected at a similar rate as in immunocompetent C57BL/6 mice. (C and D) Quantification of the BLI data from (A) and (B), respectively, normalized to the maximum radiance on day 1. (E and F) More rapid rejection of xenogeneic hESCs injected (E) i.m. and (F) i.s. but, again, delayed by ∼7 days compared with immunocompetent mice. (G and H) Quantification of the BLI data from (E) and (F), respectively, normalized to the maximum radiance on day 1. BLI data are represented as mean ± SEM from four independent experiments (n = 3).

Next, allograft responses in the *aBM* and *aBLT* models were examined in vivo against allogeneic miPSCs. Allogenized mice were injected with 1 × 10^5^ labeled miPSCs i.m. or i.s., and the survival of miPSC grafts was monitored longitudinally using BLI. The allogenized mice showed robust rejection of miPSC implanted i.m., similar to immunocompetent C57BL/6 mice, although rejection was delayed by ∼7 days compared with that observed in C57BL/6 mice (Figures 6A and 6C). In contrast, the PSC survival kinetics in the spleens of allogenized mice were indistinguishable from immunocompetent mice (Figures 6B and 6D), and no differences were observed between the *aBM* and *aBLT* models with respect to their capacity to reject allografts (Figure S6C). Allogenized mice also developed robust immune responses that resulted in rapid rejection of hESCs, although rejection of hESCs injected i.m. or i.s. was again slightly delayed by 4–7 days compared with that of immunocompetent mice (Figures 6E–6H). Overall, these findings suggest that the allogeneic thymus offers an adequate microenvironment for appropriate mouse T cell development that leads to a robust allograft response. Furthermore, these data suggest that allograft responses can be modeled in reconstituted NSG mice, although there was a delay in rejection of the PSCs at some sites in the allogenized mice compared with wild-type C57BL/6 mice.

### Allogenized Mice Demonstrate Impaired Immune Responses to Minor Histocompatibility-Mismatched Stem Cell Grafts

**Figure 7:**
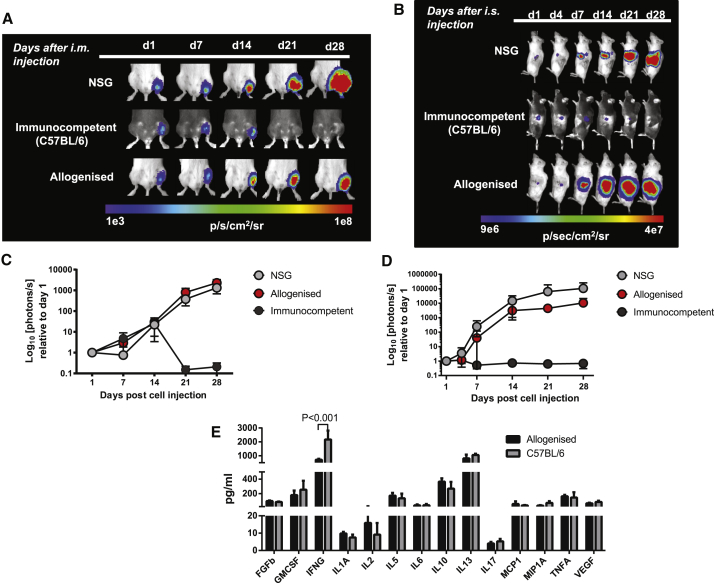
Allogenized Mice Fail to Reject mHA-Mismatched ESC Murine Allografts and Have Reduced IFN-γ Responses (A and B) Allogenized mice were unable to reject mHA-mismatched 129S1/SvImJ mESCs injected (A) i.m. (n = 5) or (B) i.s. (n = 5), and their BLI signal was similar to unmanipulated NSG mice (n = 4), whereas immunocompetent mice (n = 4, n = 5) rapidly rejected these grafts at both implantation sites. (C and D) Quantification of the BLI data from (A) and (B), respectively, normalized to the maximum radiance on day 1. (E) Significantly reduced production of IFN-γ from allogenized mouse splenocytes exposed to mHA-mismatched mESCs using a multiplex-Luminex platform. Cytokine data are represented as mean ± SEM from one experiment (n = 3).

To investigate the ability of allogenized mice to reject minor histocompatibility-mismatched grafts, we next implanted mESCs with an immunological mismatch at the minor histocompatibility antigen (mHA). The mESCs from the 129S1/SvlmJ strain (H2K^b^D^b^) were injected i.m. or i.s. into allogenized mice (donor cells from C57BL/6J mice, H2K^b^D^b^). In contrast to immunocompetent mice (C57BL/6), allogenized mice were unable to reject mHA-mismatched mESCs at either implantation site. The rate of tumor growth in allogenized mice was slightly slower than in non-engrafted NSG mice. Nevertheless, large teratomas without signs of regression developed within 30 days in allogenized and NSG mice (Figures 7A–7D).

To gain insights into the defective immune responses found in the allogenized mice in response to mHA-mismatched grafts, we next examined cytokine production of splenocytes from mHA-mismatched allogenized mice. Production of IFN-γ by splenocytes in response to short-term PMA and ionomycin stimulus was found to be significantly diminished compared with immunocompetent C57BL/6 mice (Figure 7E). Overall, the slower growth of mHA grafts in our allogenized mice shows limited effectiveness in rejecting mHA cells. However, the significant reduction of IFN-γ levels in response to PMA and ionomycin reveals an inability of the reconstituted immune system to respond appropriately to mHA grafts.

## Discussion

Preclinical trials using humanized mice could prove to be crucial in addressing the efficacy and safety of supportive therapies necessary for the maintenance of PSC grafts. One report addressed the immunogenicity of CTLA4-Ig- and PD-L1-overexpressing PSC grafts using the *hBLT* model (Rong et al., 2014). An immune response to hESCs, marked by lymphocyte infiltration and necrosis of teratomas, was observed. However, solely relying on T cell infiltration is an insufficient measure of graft rejection because infiltrates may, in fact, be suppressing the immune response (de Almeida et al., 2014). This is supported by our observation of T cell infiltration in our humanized mice that did not result in graft rejection. We therefore utilized in vivo BLI to track graft survival over time instead of relying solely on histopathology. In addition, central necrosis of tumors formed after injection of PSCs can also occur when proliferative growth is too high or too many PSCs are injected, as has been described in performing teratoma assays (Nelakanti et al., 2015).

We tested two humanized mouse models, *hSRC* and *hBLT*, to assess human immune responses to human allogeneic undifferentiated ESCs and ESC derivatives. Comparing these two mouse models, our results suggest that a functional full-scale immune response capable of rejecting human PSCs and their derivatives does not develop in these mice. We demonstrated this by first transplanting undifferentiated hESCs into *hSRC* mice. Because of the limitations of the *hSRC* mouse to properly educate human T cells in the murine thymus, we transplanted these cells in the *hBLT* model. Even though these *hBLT* mice were better reconstituted with human immune cells, the *hBLT* model still presents with abnormal reconstitution of certain subsets, such as CD8^+^ T cells as well as NK cells and other innate immune cells (Rongvaux et al., 2014). Similar to the *hSRC* model, the *hBLT* model was unable to reject miPSCs, allogeneic hESCs, differentiated hESC-ECs, and somatic HUVECs. In some *hBLT* mice, with higher numbers of intra-graft naive and regulatory T cells, graft survival even improved compared with NSG mice. Conversely, we would like to note that humanized mice have proven to be useful in the modeling of rejection of skin grafts and human islet grafts (Xiao et al., 2014). In our model, there was a strong correlation between activated immune cells and graft loss, indicating that an effective immune response to the hESC-EC grafts developed at some point. However, the limited presence of immune cells in the dLNs and allografts suggests reduced clonal expansion of effector T cells in these tissues. This was confirmed by FACS analysis and H&E staining of the dLNs, which showed limited presence of lymphocytes and macrophages as well as disorganized lymphoid structures, respectively. Both negatively influence adequate antigen presentation and subsequent stimulation of T cells.

Single-cell PCR analysis of the small percentage of activated (CD45RO^+^) lymphocytes found in the grafts, as well as splenocytes isolated from *hBLT* mice, showed a similar immunological phenotype to unstimulated PBMCs isolated from a healthy human donor, which contrasted sharply with PMA and ionomycin-stimulated healthy donor PBMCs. Even though the amount of CD45RO^+^ T cells was small in the spleens and PSC grafts of humanized mice, CD45RO^+^- and CD44^+^-expressing CD8^+^ lymphocytes in the blood were increased to ∼20%, providing evidence for a systemically activated immune system. Additional RNA-seq data, derived from *hBLT* immune blood cells at different time points during humanization, further show the development of an anergic or exhausted T cell phenotype over time after an initial activated immune profile. In vivo testing of the different immune cells from the lymphoid organs was performed after the transplant studies by re-exposing them to antigens from the transplanted cells, unencountered antigens, or a non-antigen-specific stimulus with PMA/ionomycin. However, none of the conditions described above resulted in the upregulation of pro-inflammatory cytokines and an overall inability to be activated.

We hypothesized that the education of human immune cells in the human fetal thymus failed to completely tolerize the developing human T cells to murine antigens, leading to an activated systemic immune system and a subsequent increase in morbidity and mortality in *hBLT* mice. This development of a wasting disease-like syndrome in *hBLT* mice has been described previously (Covassin et al., 2013, Greenblatt et al., 2012, Lockridge et al., 2013), but not in all laboratories (Onoe et al., 2011), and is correlated with a decrease of naive human CD45RA cells in the blood, as seen in our *hBLT* mice. The *hBLT* mice in our study were screened for chimerism 12 weeks after humanization, the transplant studies were initiated 20 weeks after humanization, and the mice were euthanized 4 weeks later. Having supporting RNA-seq and single-cell PCR data that show exhaustion of the human lymphocytes, combined with data showing impaired cytokine production during that time, indicates that there is a limited time frame for modeling human immune responses in these mice.

To test the feasibility of reconstituting an NSG mouse with a functional immune system that is capable of rejecting allogeneic PSC grafts, we next created an allogenized mouse model with allogeneic murine fetal bone marrow, thymus, and liver. This allogenized mouse model and the transplanted allogeneic hematopoietic and immune system remained viable for up to a year. Functionally, our allogenized mouse was able to fully reject the human grafts and allogeneic murine grafts. However, the allogenized mouse was unable to reject the mHA-mismatched mESCs, and these cells proliferated without signs of rejection. Cytokine profile analysis of their spleens revealed significantly lower levels of IFN-γ, which has important immunostimulatory effects and is critical for effective innate and adaptive immune responses. IFN-γ is produced predominantly by NK and NK T (NKT) cells as part of the innate immune response and by CD4^+^ T helper (Th) type 1 and CD8^+^ cytotoxic T cells when antigen-specific immunity develops. Under normal circumstances, NK and NKT cells show markedly increased IFN-γ secretion within hours after stimulation (Schoenborn and Wilson, 2007), which did not occur in our allogenized mice. The presence of a more organized lymphoid structure in the allogenized mouse compared with the humanized mouse, as well as these immune cells’ ability to be activated by allogeneic cytokines (Manz, 2007), might provide an explanation for why the allogenized mouse was able to reject allogeneic and human grafts. However, the absence of a fully functional innate immune system, as indicated by the lower levels of IFN-γ, resulted in a delayed immune response to human and allogeneic murine grafts and an inadequate response to mHA-mismatched grafts.

In summary, we have shown, with the allogenized mouse, that reconstitution of an immunodeficient mouse with a functional immune system is feasible and would allow for the modeling of PSC alloimmunity. However, current humanized mouse models suffer from inadequate reconstitution of the innate immune system and the development of a wasting disease-like syndrome that renders them inadequate for long-term PSC transplant studies. To limit the development of this syndrome, future efforts will focus on genetic modification of the SIRPA-CD47 pathway (i.e., providing a “don’t eat me signal”) (Lavender et al., 2013) as well as the continuing development of NSG mice that transgenically express HLA molecules in their thymus (Babad et al., 2015, Shultz et al., 2010), therefore allowing HLA-restricted T cell selection for both human and murine antigens. Moreover, NSG mice expressing important human cytokines for innate and adaptive immune responses, such as macrophage colony-stimulating factor (M-CSF), IL-3/granulocyte macrophage colony-stimulating factor (GM-CSF), and TPO, already exist (Rongvaux et al., 2014), and combination of one or more properties of these mice could result in a more effective model with which to conduct studies of the immunobiology of PSC therapeutics.

## Experimental Procedures

### Humanized and Allogenized Mouse Development

All experiments were performed with approval of the Animal Care and Use Committee at Stanford University and the Institutional Animal Care and Use Committee (IACUC) committee at University of Massachusetts Medical School. Humanized *hSRC* mice (12 weeks old) were developed by γ-irradiating, 100 centigray (cGy), NOD.Cg-*Prkdc*^*scid*^ *IL2rg*^*tm1Wj1*^/Sz (NSG) mice (3-4 weeks old) and transplanting them with lineage depleted HSCs (100,000 CD34^+^ HSC). Humanized *hBLT* mice (12 weeks old; male) were developed by γ-irradiating (200 cGy) NSG mice (6–8 weeks old), surgically implanting HLA-A2^neg^ human fetal liver and thymus under the kidney capsule, and injecting autologous HSCs (100,000 CD34^+^ HSC). All mice were screened for human cell chimerism levels 12 weeks post-engraftment. Allogenized mice were generated at Stanford University using a similar methodology as that used for generating *hBLT* mice (Supplemental Experimental Procedures). Control female mice, FVB/NJ mice, and C57BL/6J mice were purchased from The Jackson Laboratory at 6–8 weeks of age and aged to match our humanized and allogenized mice.

### In Vivo BLI

Survival of transplanted hESC-ECs was longitudinally monitored with BLI using the Xenogen In Vivo Imaging System (Caliper Life Sciences). After i.p. injection of the reporter probe D-luciferin (375 mg/kg body weight), the mice were placed in the light-tight chamber and imaged with integration times of 5 s to 2 min, depending on emission intensity. Quantification of the BLI signal was performed by using the maximum photons per square centimeter and is presented as Log10 (photons s-1).

### Flow Cytometric Analysis of Graft-Infiltrating Lymphocytes

Cells were isolated from s.c. and i.m. injected grafts and resuspended in FACS buffer (phosphate-buffered saline [PBS] containing 2% fetal bovine serum [FBS] and 2 mM EDTA), and the Fc receptor was blocked by anti-human Fc receptor-blocking antibody (Miltenyi Biotec). Samples were then stained with fluorophore-conjugated monoclonal antibodies against murine CD45 and the following human markers: CD45, CD3, CD4, CD8, CD25, FoxP3, CD11b, and NK1.1 (1:100; BD Biosciences, eBioscience, BioLegend, and Thermo Fisher). Cells were fixed and permeabilized using BD Cytofix/Cytoperm fixation, and intracellular staining was performed. Cells were assayed using an LSRII flow cytometer (BD Biosciences) and further analyzed with FlowJo software (Tree Star, Ashland, OR, USA).

### RNA-Seq

CD45^+^ and CD3^+^ cells were isolated by flow cytometry using a FACSAria II special order research product (SORP) flow cytometer (BD Biosciences). Total RNA was extracted and quantified using the miRNeasy Kit (QIAGEN) according to the manufacturer’s protocol. Ten nanograms of total RNA was used to generate index-tagged paired-end cDNA libraries. Briefly, mRNAs were purified and then amplified to cDNA using the Ovation RNA-Seq System V2 (NuGEN). Libraries were prepared and indexed using the NEBNext Ultra DNA Library Prep Kit for Illumina and NEBNext Multiplex Oligos. Sequencing was performed with Illumina’s HiSeq4000 platform using paired-end reads at an average length of 100 bp (2 × 100).

### RNA-Seq Analysis

The sequenced reads were aligned to the human genome (hg19, downloaded from University of California, Santa Cruz [UCSC]) by HISAT2 (https://ccb.jhu.edu/software/hisat2/index.shtml; Pertea et al., 2016). We then used FeatureCounts (http://bioinf.wehi.edu.au/featureCounts/; Liao et al., 2014) to quantitate the transcriptome with genome annotation GENCODE 19 (hg19, version 19). DESeq2 (https://bioconductor.org/packages/release/bioc/html/DESeq2.html; Love et al., 2014) of Bioconductor was applied to the raw reads of the transcriptome to normalize and generate the table of differentially expressed genes (DEGs) (likelihood ratio test [LRT], p < 0.05). Hierarchical clustering was then implemented on the DEGs. Function enrichment analyses of the DEG clusters were then implemented by GeneAnswers from Bioconductor (https://www.bioconductor.org/packages/release/bioc/html/GeneAnswers.html).

### Single-Cell PCR Analysis

Gene expression of single cells was done using a 96.96 Dynamic Array chip (M96, Fluidigm). Single cells were sorted into each well of a 96-well PCR plate. After cell sorting and brief PCR plate centrifugation, the plate was placed in a thermocycler for reverse transcription into cDNA, followed by pre-amplification for 18 cycles. Finally, samples and assays were loaded in the M96 Fluidigm plate using a NanoFlex integrated fluidic circuits (IFC) controller (Fluidigm), followed by real-time PCR in the BioMark high-definition (HD) system (Fluidigm). The results were analyzed using the Fluidigm real-time PCR analysis software.

### Multiplex-Luminex Cytokine Assay

Production of various cytokines was measured in cell culture supernatant using a multiplex-Luminex (LabMap200 system, Luminex) together with Panomics antibodies at the Human Immune Monitoring Center at Stanford University.

### Statistical Methods

Statistical tests were performed using GraphPad Prism software. Bar graphs represent the mean and SEM for each group. R package FactoMineR (http://cran.r-project.org/web/packages/FactoMineR/index.html) was applied for PCA and visualization of gene expression. The statistical method used in Figure 3 for the correlation analysis was a Pearson correlation test. In Figure 7E, the mean levels of IFN-γ between two groups were compared using an unpaired Student’s t test. The engraftment data in Figure S3B were analyzed by comparing the mean percentage of cells between two groups using multiple t tests.

## Author Contributions

N.G.K., P.E.d.A., and J.P.S. conceived, performed, and interpreted the experiments and wrote the manuscript. R.V.N. assisted with cell implantation and BLI. R.J.S. and S.D. assisted with ESC differentiation. V.S.F. conducted the gene expression analysis by Fluidigm. N.Y.S. performed biostatistical analyses on the single-cell PCR and RNA-seq data. E.M. performed immunofluorescence staining of tissue grafts. C.L. assisted with flow cytometry sample processing and data acquirement. A.J.C. performed histology analysis on tissue samples. M.A.B. and D.L.G. provided the humanized mice, experimental advice, and manuscript writing. L.D.S. provided experimental advice and manuscript writing. J.C.W. conceived study, provided experimental advice, manuscript writing, and funding support.

## Accession Numbers

The accession number for the raw and processed data from the next-generation RNA-seq experiments reported in this paper is NCBI GEO: GSE100791.

## Supplemental Information

Document S1. Figures S1–S7 and Tables S1 and S2

Document S2. Article plus Supplemental Information
