# Supplementary material for: Alloimmune Responses of Humanized Mice to Human Pluripotent Stem Cell Therapeutics
Source: Cell Rep. 2017 Aug 22;20(8):1978–90. doi: 10.1016/j.celrep.2017.08.003 (PMC5573767; doi:10.1016/j.celrep.2017.08.003)
Supplement: Document S1. Figures S1–S7 and Tables S1 and S2 [file mmc1.pdf]

Cell Reports, Volume *20*

Supplemental Information

Alloimmune Responses of Humanized Mice

to Human Pluripotent Stem Cell Therapeutics

Nigel G. Kooreman, Patricia E. de Almeida, Jonathan P. Stack, Raman V. Nelakanti, Sebastian Diecke, Ning-Yi Shao, Rutger-Jan Swijnenburg, Veronica Sanchez-Freire, Elena Matsa, Chun Liu, Andrew J. Connolly, Jaap F. Hamming, Paul H.A. Quax, Michael A. Brehm, Dale L. Greiner, Leonard D. Shultz, and Joseph C. Wu

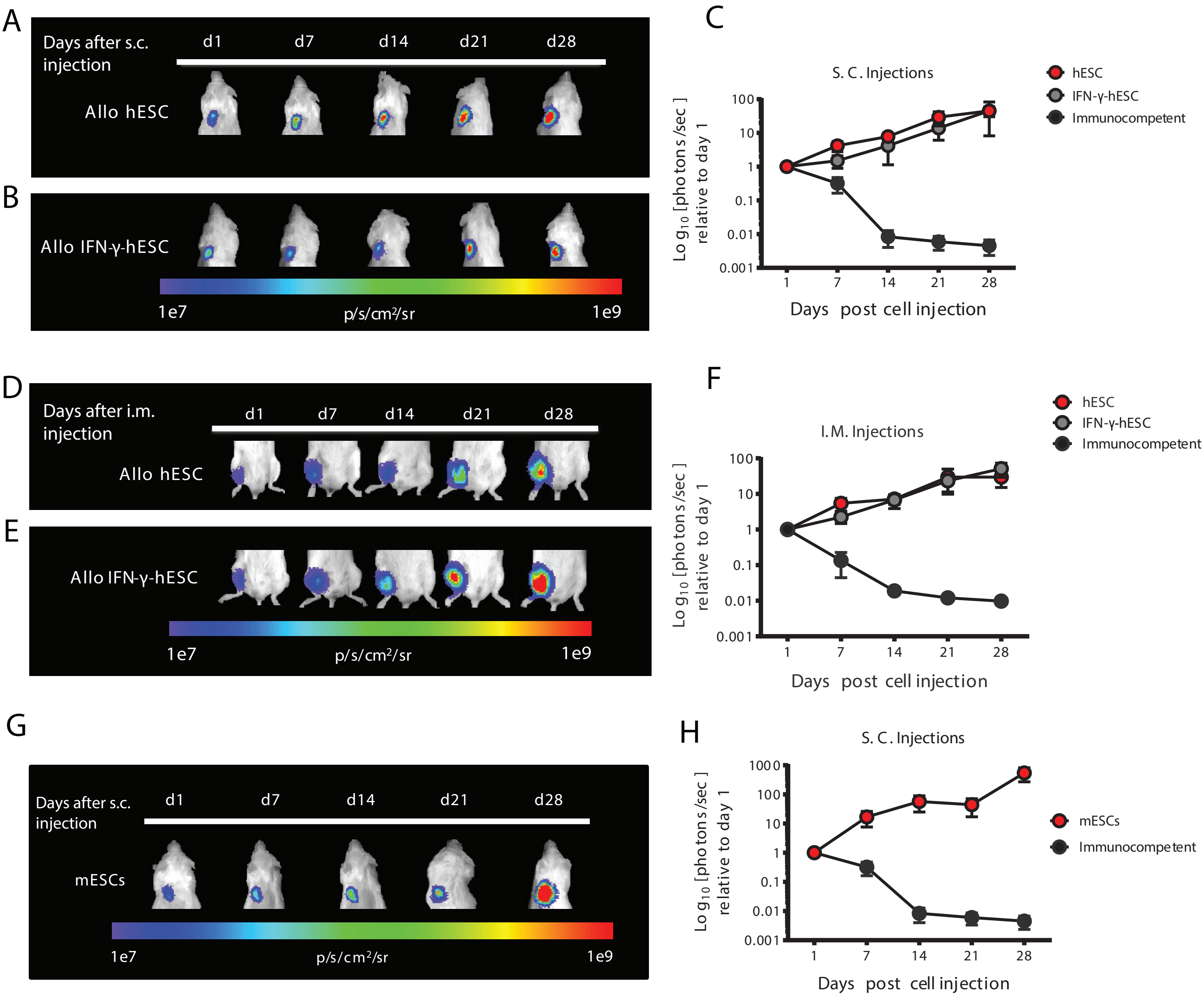
A
Days after s.c. injection
d1
d7
d14
d21
d28
Allo hESC
B
Allo IFN-γ-hESC
1e7
p/s/cm²/sr
1e9
C
S. C. Injections
hESC
IFN-γ-hESC
Immunocompetent
Log10 [photons/sec] relative to day 1
Days post cell injection
D
Days after i.m. injection
Allo hESC
E
Allo IFN-γ-hESC
F
I.M. Injections
G
mESCs
H
mESCs
Immunocompetent

Figure S1

Figure S1, Related to Figure 1. *hSRC* mice fail to reject allogeneic unstimulated, IFN-γ-stimulated hESCs and murine ESCs. (A) hESCs (*Allo hESCs*) and (**B)** IFN-γ-stimulated hESCs (*IFN-Allo hESCs*), implanted subcutaneously (s.c.), proliferated and showed no sign of immunological rejection as assessed by BLI. (**C**) Quantification of BLI data for s.c. injections of (A) allogeneic hESCs (n=10) and (B) IFN-γ-hESCs (n=8) in *hSRC* model and immunocompetent C57BL/6 mice (n=5). (**D)** hESCs (*Allo hESCs*) and (**E)** IFN-γ-stimulated hESCs (*IFN-Allo hESCs*), implanted intramuscularly (i.m.), proliferated and showed no sign of immunological rejection as assessed by BLI. (**F**) Quantification of BLI data for i.m. injections of hESCs (n=10) and IFN-γ-hESCs (n=8) in *hSCR* model and immunocompetent C57BL/6 mice (n=3) (*Figure 6)*. Quantitative data are represented as mean ± SEM. **(G)** mESCs (129S1/SvJ strain; *mESCs*), implanted s.c., proliferated and were not rejected by *hSRC* mice (n=4) as assessed by BLI. In contrast, immunocompetent (C57BL/6) mice (n=5) rejected allogeneic (129S1/SvJ) mESCs within 2 weeks after cell implantation. (**H)** Quantification of the BLI data from (G) normalized to the maximum radiance at day 1. Data are represented as mean ± SEM.

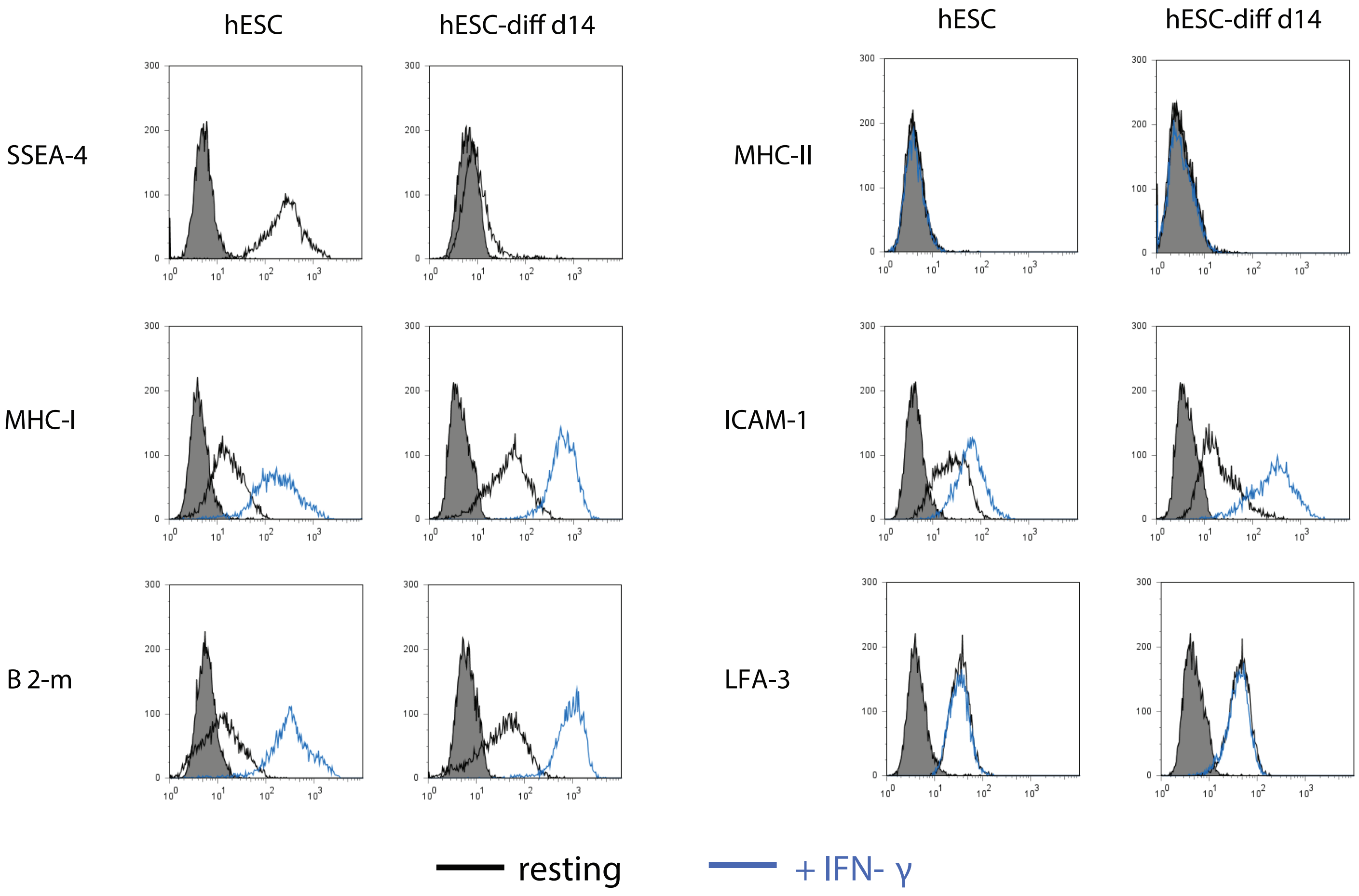
hESC
hESC-diff d14
hESC
hESC-diff d14
SSEA-4
MHC-II
MHC-I
ICAM-1
B 2-m
LFA-3
resting
+ IFN- γ

Figure S2

Figure S2, Related to Figure 2. IFN-γ stimulation and differentiation of hESCs increases MHC-I expression and potential immunogenicity. FACS analysis of $1x10^6$ hESCs after 48 hr *in vitro* IFN-γ stimulation (blue line) increases cell surface expression of MHC-I, B2-m and ICAM-1 compared to the resting state (black line). A similar trend of upregulation of these molecules is seen after 14 days of differentiation (hESC-diff D14, n=20 EBs). Reduction in expression of the pluripotency marker SSEA-4 at day 14 of EB formation indicates adequate induction of differentiation.

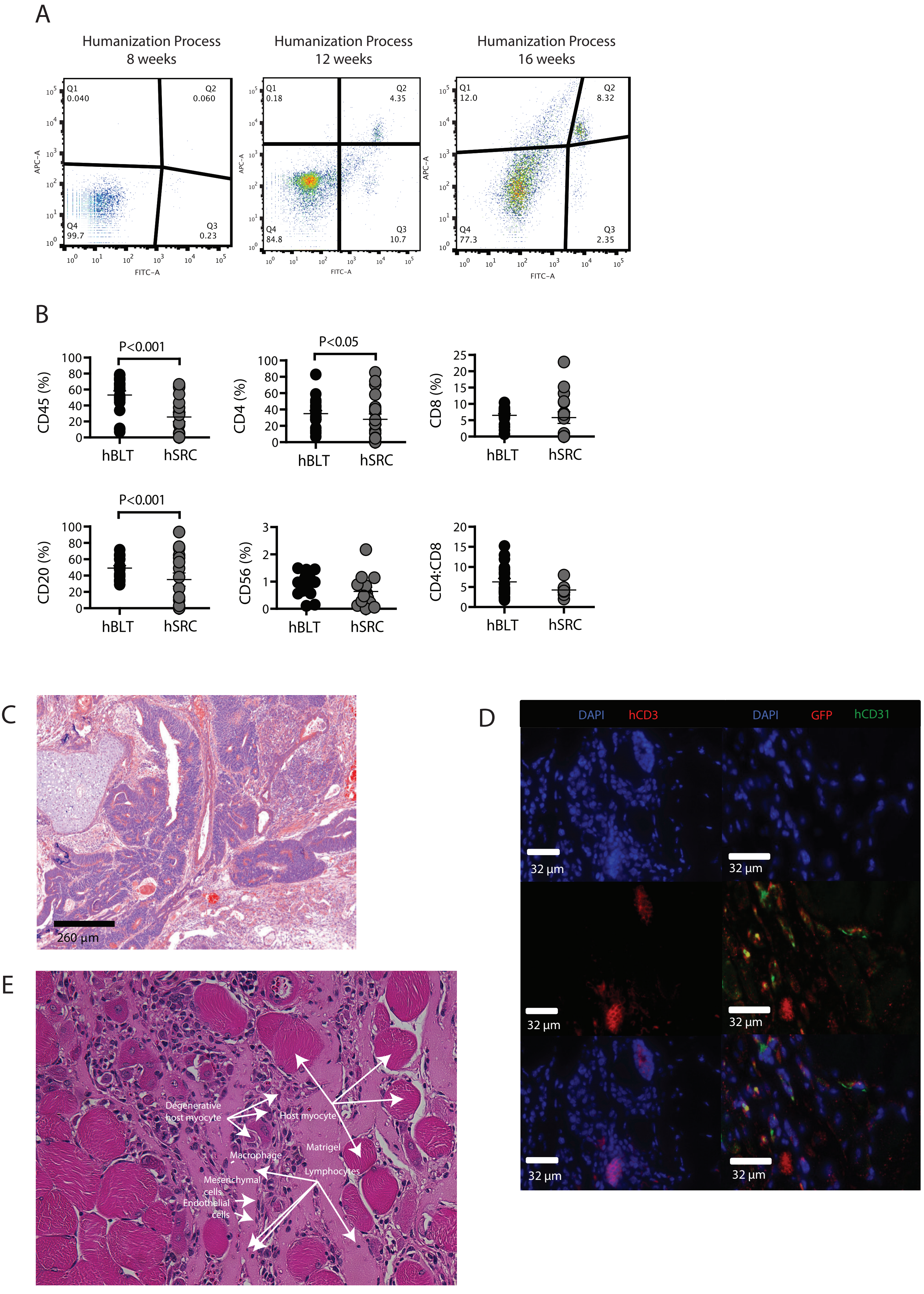
A
Humanization Process
8 weeks
Humanization Process
12 weeks
Humanization Process
16 weeks
Q1 0.040
Q2 0.060
Q4 99.7
Q3 0.23
Q1 0.18
Q2 4.35
Q4 84.8
Q3 10.7
Q1 12.0
Q2 8.32
Q4 77.3
Q3 2.35
APC-A
FITC-A
B
P<0.001
P<0.05
CD45 (%)
CD4 (%)
CD8 (%)
CD20 (%)
CD56 (%)
CD4:CD8
hBLT
hSRC
C
260 µm
D
DAPI
hCD3
DAPI
GFP
hCD31
32 µm
E
Degenerative host myocyte
Host myocyte
Macrophage
Matrigel
Lymphocytes
Mesenchymal cells
Endothelial cells

Figure S3

Figure S3, Related to Figure 2, 3. Superior engraftment of human immune cell subsets in *hBLT* mice did not result in the rejection of hESCs and their derivatives. (**A)** Beginning at 8 weeks after engraftment of human fetal bone marrow, liver and thymus, the presence of human immune cells (CD3+: *FITC,* CD45+: *APC)* can be detected in the *hBLT* circulation at 12 weeks. (**B)** Percentage of human CD45+ immune cells and the proportion of each human cell subset of human CD45+ cells in the peripheral blood 12 weeks after human immune cell engraftment of *hSRC* (n=18) and *hBLT* (n=21). Analysis was performed prior to the transplantation of hESCs. Data are presented as individual mice (dots) with mean ± SEM. **(C)** Representative H&E image of teratoma developed in the spleen of *hBLT* mouse 4 weeks after hESC transplantation. (**D)** Grafts isolated from *hBLT* mice 4 weeks after hESC-EC transplantation shows presence of human $CD3^+$ cells (hCD3; red) (left panel) and hESC-ECs (right panel) by co-staining for nuclei (blue), anti-GFP (red), and anti-human CD31 (hCD31; green). (**E)** Analysis of the Matrigel plugs reveals very little immune cell activity with a low presence of T-cells and macrophages. Even in comparison to a syngeneic transplant setting, the numbers are very low. Within the grafts the presence of spindle shaped cells that were forming tubes is indicative of endothelial cells, as confirmed by the immunohistochemistry staining in *Figure S3D*.

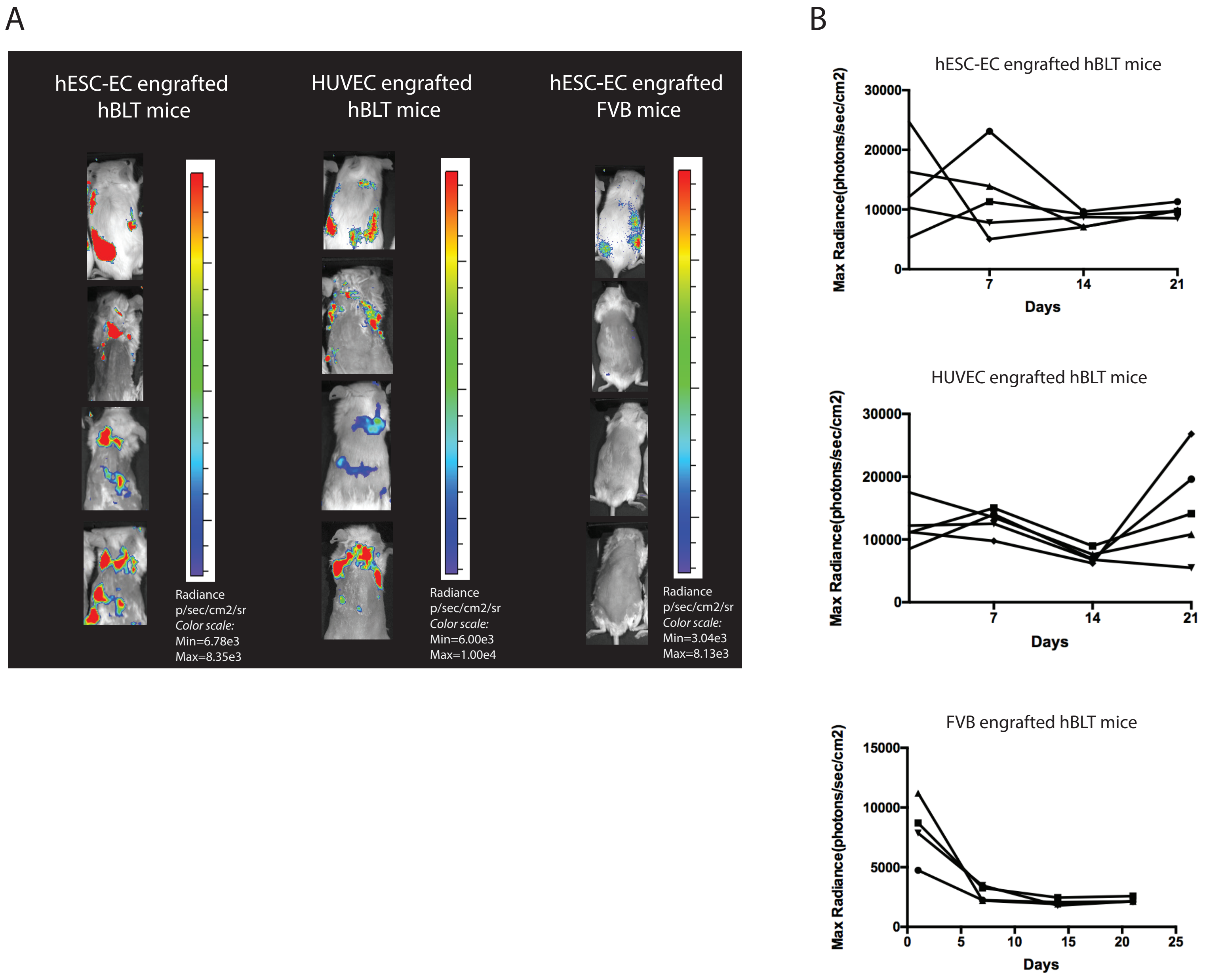

A
hESC-EC engrafted hBLT mice
HUVEC engrafted hBLT mice
hESC-EC engrafted FVB mice
Radiance p/sec/cm2/sr Color scale: Min=6.78e3 Max=8.35e3
Radiance p/sec/cm2/sr Color scale: Min=6.00e3 Max=1.00e4
Radiance p/sec/cm2/sr Color scale: Min=3.04e3 Max=8.13e3
B
hESC-EC engrafted hBLT mice
HUVEC engrafted hBLT mice
FVB engrafted hBLT mice
Max Radiance(photons/sec/cm2)
Days

Figure S4

Figure S4, Related to Figure 2, 3. *hBLT* mice demonstrate an impaired ability to reject grafts derived from both somatic cells and ESCs. (A) Luciferase-labeled hESC-derived endothelial cells (hESC-ECs) (*left panel)* and HUVEC cells (*middle panel)* engrafted into humanized mice show graft persistence and proliferation over time, whereas hESC-ECs engrafted in immunocompetent FVB mice (*right panel*) are rejected within one week. **(B)** Quantification of the BLI signal (max. radiance) of the transplanted cells displayed in *Figure S4A* at day 7, 14 and 21.

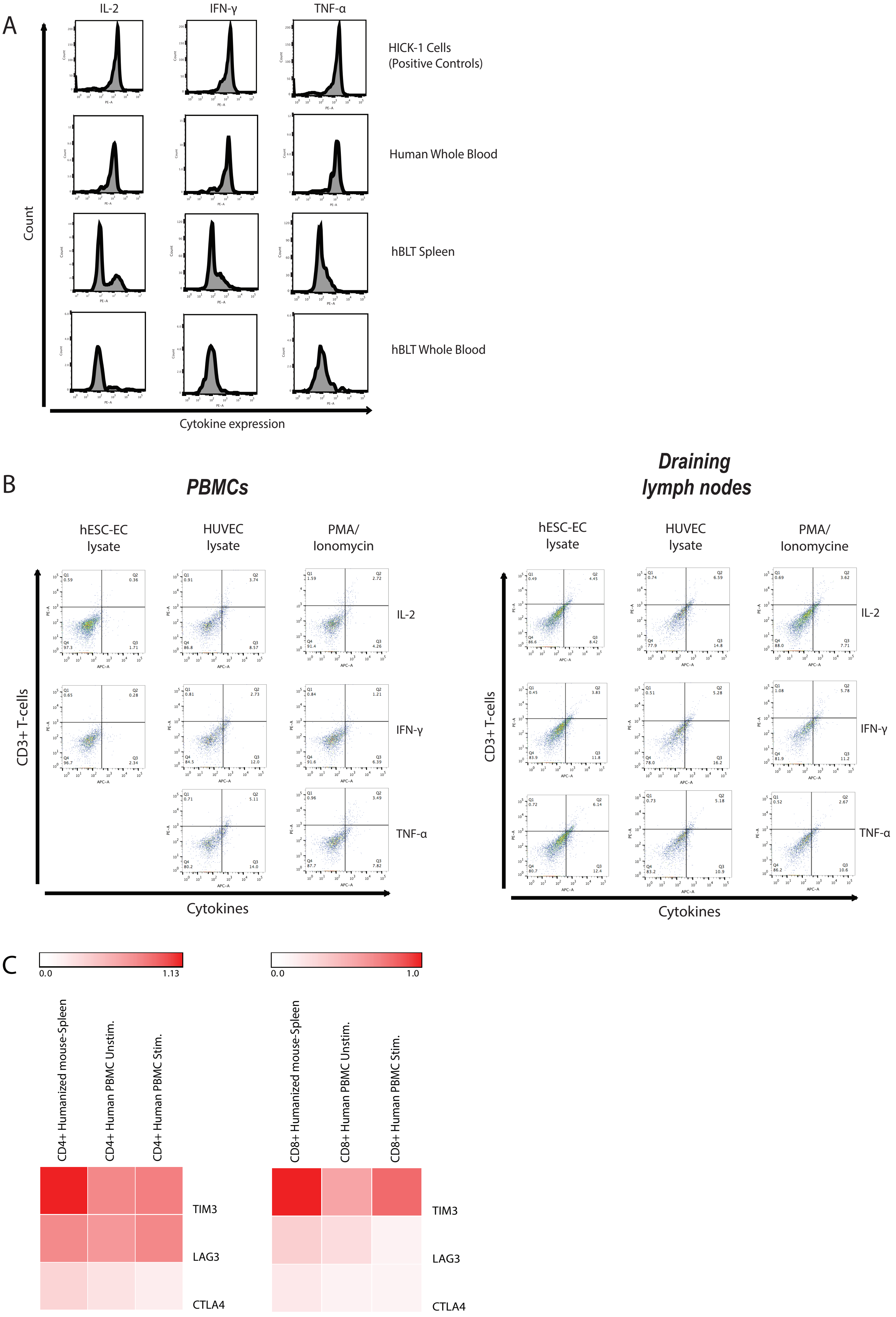
A
IL-2
IFN-γ
TNF-α
HICK-1 Cells
(Positive Controls)
Human Whole Blood
hBLT Spleen
hBLT Whole Blood
Count
Cytokine expression
B
PBMCs
Draining
lymph nodes
hESC-EC
lysate
HUVEC
lysate
PMA/
Ionomycin
PMA/
Ionomycine
IL-2
IFN-γ
TNF-α
CD3+ T-cells
Cytokines
C
0.0
1.13
1.0
CD4+ Humanized mouse-Spleen
CD4+ Human PBMC Unstim.
CD4+ Human PBMC Stim.
CD8+ Humanized mouse-Spleen
CD8+ Human PBMC Unstim.
CD8+ Human PBMC Stim.
TIM3
LAG3
CTLA4

Figure S5

Figure S5, Related to Figure 4. Inability of the *hBLT* immune cells to mount an effective immune response and high expression of T-cell anergy/exhaustion genes. (A) Human immune cells isolated from *hBLT* mice are incapable of mounting a pro-inflammatory immune response with IL2, IFN-γ and TNF-α release. Peripheral human immune cells (*hBLT Whole blood)* in 16-week old *hBLT* mice are anergic to PMA/Ionomycin stimulation. In contrast, splenic human immune cells (*hBLT Spleen)* reveal a limited response, but this response is limited as compared to the control samples (*HiCK-1; Human whole blood).* Data representative of three experiments. **(B)** Human immune cells isolated from Matrigel plugs of hESC-EC engrafted mice show low cytokine production upon stimulation with hESC-EC lysate. These results were comparable to stimulation with an antigen (HUVEC lysate) the immune cells had not been previously exposed to. Even upon non-antigen specific positive control stimulation with PMA/Ionomycin, hESC-EC engrafted human immune cells from the Matrigel plugs show very limited pro-inflammatory cytokine production. Similar results are found when performing these experiments on human immune cells isolated from lymph nodes from *hBLT* mice. In these mixed-lymphocyte reactions the percentage of cytokine producing immune cells is limited, especially when comparing the results to the positive control sample of whole human blood in *Figure S5A.* PE: (Cytokines, APC: CD3). (**C**) Splenocytes isolated from *hBLT* mice (n=12), 4 weeks after transplantation of hESC-ECs, show upregulation of *TIM3, LAG3* and *CTLA4* genes in $CD4^+$ and $CD8^+$ lymphocytes compared to unstimulated and PMA/Ionoycin stimulated control human PBMCs (n=4).

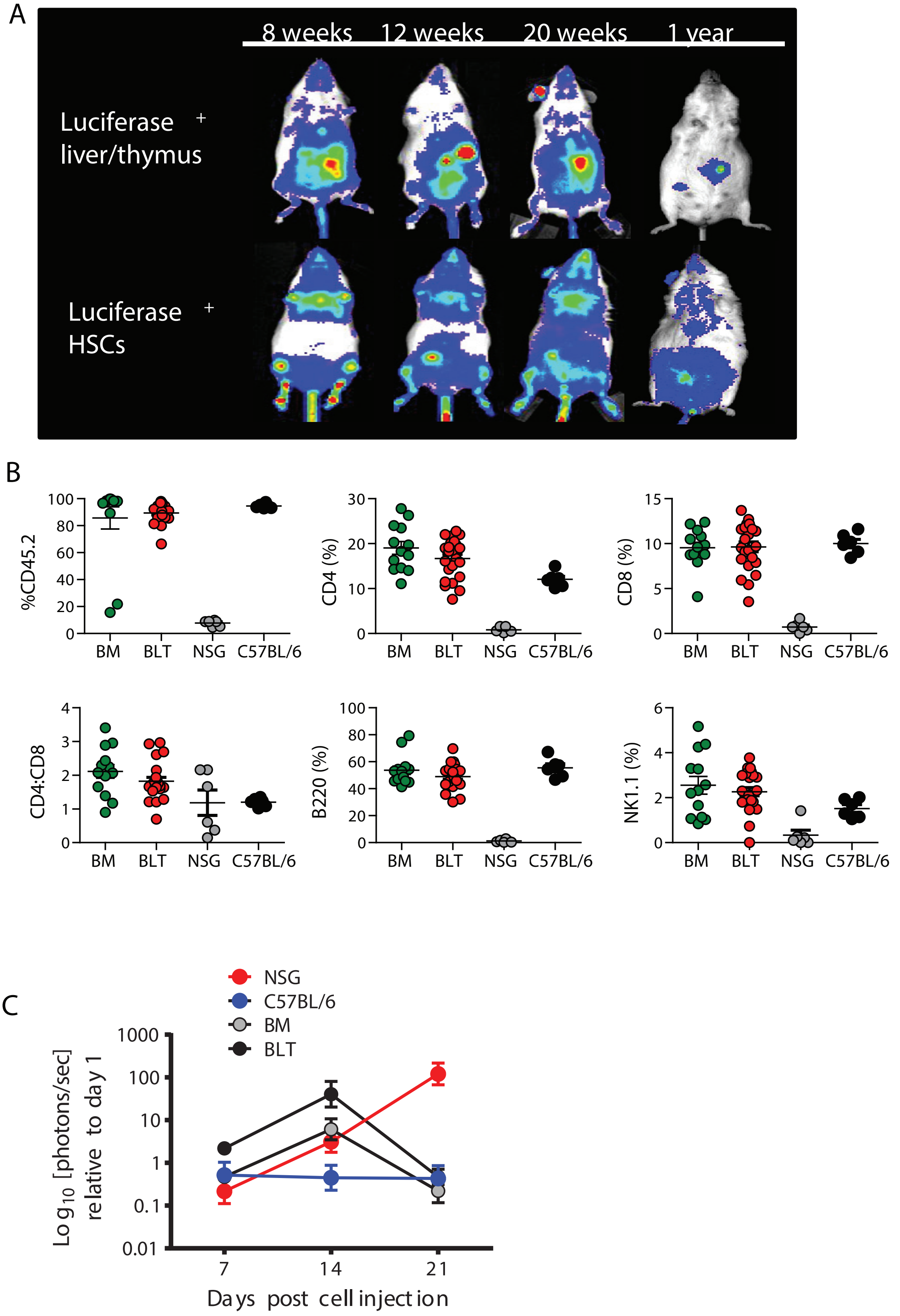
A
8 weeks
12 weeks
20 weeks
1 year
Luciferase + liver/thymus
Luciferase + HSCs
B
%CD45.2
CD4 (%)
CD8 (%)
CD4:CD8
B220 (%)
NK1.1 (%)
BM
BLT
NSG
C57BL/6
C
NSG
C57BL/6
BM
BLT
Log10 [photons/sec] relative to day 1
Days post cell injection
7
14
21
1000
100
10
1
0.1
0.01

Figure S6

Figure S6, Related to Figure 5, 6, 7. Allogenised *BLT* mice show sustained viability of grafted donor tissue, high levels of immune cell engraftment, and similar functionality between *BM* and *BLT* models. (A) BLI demonstrating sustained engraftment of luciferase positive allogeneic immune cells in bone marrow compartments and sustained viability of luciferase positive allogeneic liver and thymus transplanted in the abdomen of NSG mice. **(B)** Similar engraftment of C57BL/6 total leukocytes (CD45.2), helper and cytotoxic ($CD8^+$) T cells, B cells ($B220^+$), and NK cells ($NK1.1^+$) in *mBM* (n=13) and *mBLT* (n=23) NSG models compared to wild-type C57BL/6 mice (n=6). **(C)** Quantification of BLI data relative to the maximum radiance at day 1 shows that *BM* (n=3) and *BLT* (n=3) allogenised mice have similar effectiveness in the rejection of miPSC allografts (n=3), although the rate of rejection was delayed by ~7 days compared to wild-type C57BL/6 mice (n=3). Data are presented as individual mice (dots) with mean ± SEM.

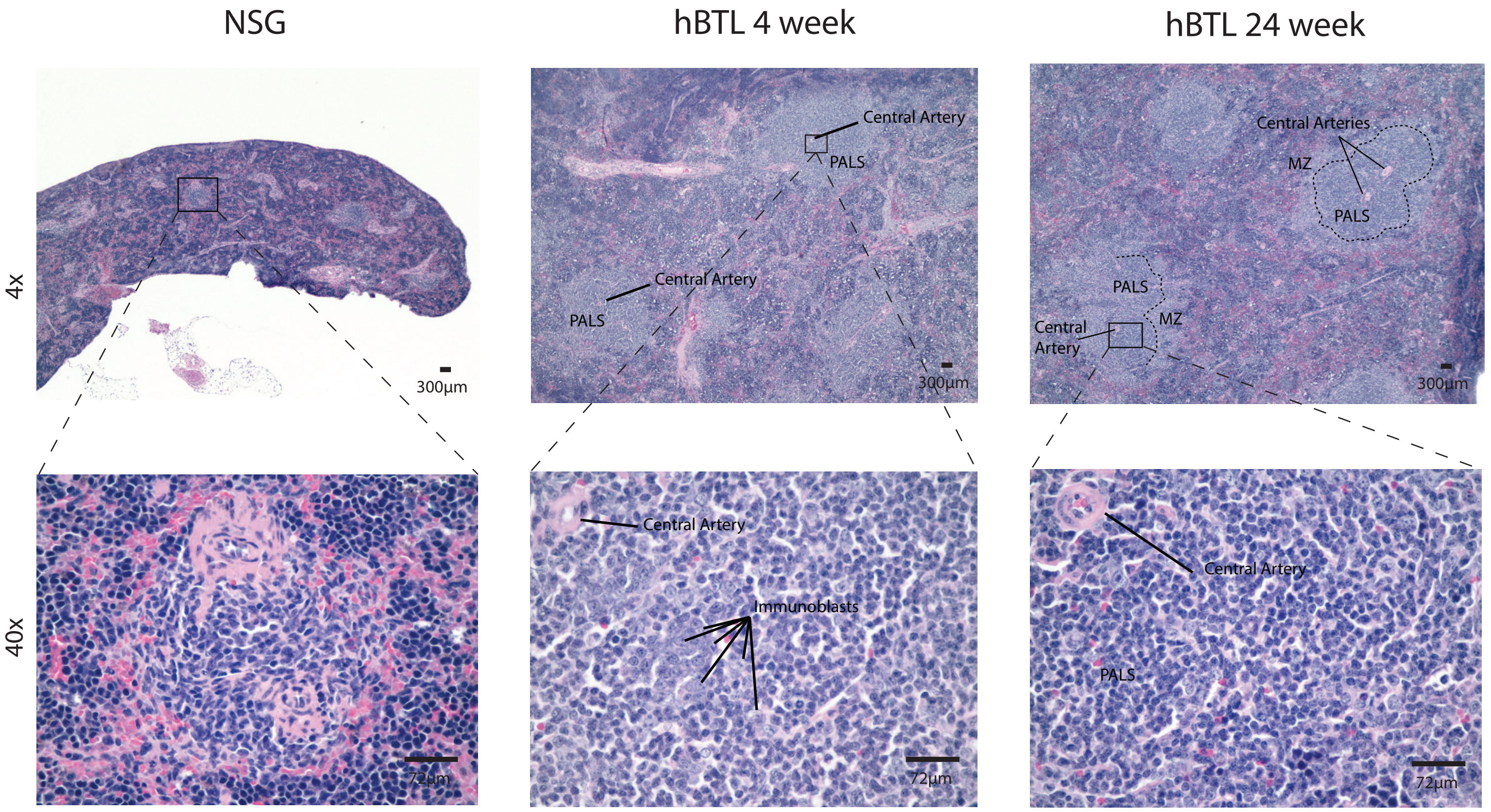
NSG
hBTL 4 week
hBTL 24 week
4x
40x
300µm
Central Artery
PALS
Central Artery
PALS
300µm
Central Arteries
MZ
PALS
PALS
MZ
Central
Artery
300µm
72µm
Central Artery
Immunoblasts
72µm
Central Artery
PALS
72µm

Figure S7

Figure S7, Related to Figure 5. Older *hBLT* mice have a more mature splenic architecture compared to young *hBLT* mice. Compared to the NSG background mouse strain, *hBLT* mice at one week after humanization have abundant periarteriolar lymphatic sheaths (PALS), comprised of mononuclear cells. Some of these are small lymphocytes interspersed with clumps of larger cells, likely to be immunoblasts, as well as the lack of a marginal zone (MZ). Both aspects indicating an immature architecture. By week 24 after humanization, the numbers of periarteriolar small lymphocytes have increased and a marginal zone containing macrophages surrounds them, indicating maturation.

Table S1: List of antibodies used in FACS

Antibody	Fluorophore Conjugate	Company
CD3	APC	BD Bioscience
CD3	APC	ThermoFisher
CD4	Pe-Cy7	BioLegend
CD8	FITC	BD Bioscience
CD45RA	PE	BioLegend
CD45RO	Pacific Blue	BioLegend
mouse CD45.1	PerCP/Cy5.5	BD Bioscience
human CD45	Pacific Orange	Life technologies
human CD45	PerCP	ThermoFisher
FoxP3	FITC	eBioscience
CD56	FITC	BioLegend
CD20	Pe-Cy7	BD Bioscience
CD44	Pacific Blue	BioLegend
CD11b	APC	eBioscience
Fixable Viability Dye	AlexaFluor780	Life Technologies

Table S1, Related to Figure 3. FACS antibody panel used for analyzing and sorting human PBMC samples isolated from *hBLT* mice.

Table S2: Human Primer sequences used in the single-cell PCR analysis

	Gene	Forward Primer	Reverse Primer
1	BCL2L1	GCCACTTACCTGAATGACCACC	AACCAGCGGTTGAAGCGTTCCT
2	CCL11	GCTACAGGAGAATCACCAGTGG	GGAATCCTGCACCCACTTCTTC
3	CCL5	CCTGCTGCTTTGCCTACATTGC	ACACACTTGGCGGTTCTTTCGG
4	CCL7	ACAGAAGGACCACCAGTAGCCA	GGTGCTTCATAAAGTCCTGGACC
5	CCR2	CAGGTGACAGAGACTCTTGGGA	GGCAATCCTACAGCCAAGAGCT
6	CCR3	TACTCCCTGGTGTTCACTGTGG	ACGAGGAAGAGCAGGTCCGAAA
7	CCR4	CTCTGGCTTTTGTTCACTGCTGC	AGCCCACAGTATTGGCAGAGCA
8	CCR5	TCTCTTCTGGGCTCCCTACAAC	CCAAGAGTCTCTGTCACCTGCA
9	CCR7	CAACATCACCAGTAGCACCTGTG	TGCGGAACTTGACGCCGATGAA
10	CD247	CTGCCTTGTTCCTGAGAGTGAAG	CATCGTACTCCTCTCTTCGTCC
11	CD27	ACTACTGGGCTCAGGGAAAGCT	GGATCACACTGAGCAGCCTTTC
12	CD28	GAGAAGAGCAATGGAACCATTATC	TAGCAAGCCAGGACTCCACCAA
13	CD3G	GCATTTTCGTCCTTGCTGTTGGG	GGTCATCTTCTCGATCCTTGAGG
14	CD4	CCTCCTGCTTTTCATTGGGCTAG	TGAGGACACTGGCAGGTCTTCT
15	CD40LG	GCGGCACATGTCATAAGTGAGG	GTCCTTGTCTTTTAACGGTCAGC
16	CD8	ACTTGTGGGGTCCTTCTCCTGT	TGTCTCCCGATTTGACCACAGG
17	CD80	CTCTTGGTGCTGGCTGGTCTTT	GCCAGTAGATGCGAGTTTGTGC
18	CD86	CCATCAGCTTGTCTGTTTCATTCC	GCTGTAATCCAAGGAATGTGGTC
19	CEBPB	AGAAGACCGTGGACAAGCACAG	CTCCAGGACCTTGTGCTGCGT
20	CREBBP	AGTAACGGCACAGCCTCTCAGT	CCTGTCGATACAGTGCTTCTAGG
21	CSF2	GGAGCATGTGAATGCCATCCAG	CTGGAGGTCAAACATTTCTGAGAT
22	CTLA4	ACGGGACTCTACATCTGCAAGG	GGAGGAAGTCAGAATCTGGGCA
23	CXCR3	ACGAGAGTGACTCGTGCTGTAC	GCAGAAAGAGGAGGCTGTAGAG
24	CXCR4	CTCCTCTTTGTCATCACGCTTCC	GGATGAGGACACTGCTGTAGAG
25	FASLG	GGTTCTGGTTGCCTTGGTAGGA	CTGTGTGCATCTGGCTGGTAGA
26	GATA3	ACCACAACCACACTCTGGAGGA	TCGGTTTCTGGTCTGGATGCCT
27	GFI1	GCTTCAAGAGGTCATCCACACTG	ACCTGGCACTTGTGAGGCTTCT
28	GLMN	GCTGGAATCTCGTTGGTCCTGT	GCTCTTCAATCAGTTCAAGCAAAC
29	GPR44	TGGAGTCATCCTCTTCGTGGTG	AGTAGGTGAAGAAGGGCAGGGA
30	HAVCR2	GACTCTAGCAGACAGTGGGATC	GGTGGTAAGCATCCTTGGAAAGG
31	ICOS	CCCATAGGATGTGCAGCCTTTG	GGCTGTGTTCACTGCTCTCATG
32	IFNG	GAGTGTGGAGACCATCAAGGAAG	TGCTTTGCGTTGGACATTCAAGTC
33	IGSF6	GCGAGAGCTAAACAGACAGGAG	CGGTCACATAGACAGAGAGCAG
34	IL10	TCTCCGAGATGCCTTCAGCAGA	TCAGACAAGGCTTGGCAACCCA
35	IL12B	GACATTCTGCGTTCAGGTCCAG	CATTTTTGCGGCAGATGACCGTG
36	IL12RB2	AGACCTCAGTGGTGTAGCAGAG	TGATGACCAGCGGTTCAGGATC
37	IL13	ACGGTCATTGCTCTCACTTGCC	CTGTCAGGTTGATGCTCCATACC
38	IL13RA1	CCTGAATGAGAGGATTTGTCTGC	CAGTCACAGCAGACTCAGGATC
39	IL15	AACAGAAGCCAACTGGGTGAATG	CTCCAAGAGAAAGCACTTCATTGC
40	IL17A	CGGACTGTGATGGTCAACCTGA	GCACTTTGCCTCCCAGATCACA
41	IL18	GATAGCCAGCCTAGAGGTATGG	CCTTGATGTTATCAGGAGGATTCA
42	IL18R1	GGAGGCACAGACACCAAAAGCT	AGGCACACTACTGCCACCAAGA
43	IL1R1	GTGCTTTGGTACAGGGATTCCTG	CACAGTCAGAGGTAGACCCTTC
44	IL1R2	GGCTATTACCGCTGTGTCCTGA	GAGAAGCTGATATGGTCTTGAGG
45	IL2	AGAACTCAAACCTCTGGAGGAAG	GCTGTCTCATCAGCATATTCACAC
46	IL23A	GAGCCTTCTCTGCTCCCTGATA	GACTGAGGCTTGGAATCTGCTG
47	IL2RA	GAGACTTCCTGCCTCGTCACAA	GATCAGCAGGAAAACACAGCCG
48	IL4	CCGTAACAGACATCTTTGCTGCC	GAGTGTCCTTCTCATGGTGGCT
49	IL5	GGAATAGGCACACTGGAGAGTC	CTCTCCGTCTTTCTTCTCCACAC
50	IL6	AGACAGCCACTCACCTCTTCAG	TTCTGCCAGTGCCTCTTTGCTG

51	IL7	GACAGCATGAAAGAAATTGGTAGC	CAACTTGCGAGCAGCACGGAAT
52	IL9	GACCAGTTGTCTCTGTTTGGGC	TTTCACCCGACTGAAAATCAGTGG
53	INHA	TCCCAAGCCATCCTTTTCCCAG	TCACCTGGCGGCTGCGTGTAT
54	IRF1	GAGGAGGTGAAAGACCAGAGCA	TAGCATCTCGGCTGGACTTCGA
55	IRF4	GAACGAGGAGAAGAGCATCTTCC	CGATGCCTTCTCGGAACTTTCC
56	JAK1	GAGACAGGTCTCCCACAAACAC	GTGGTAAGGACATCGCTTTTCCG
57	JAK2	CCAGATGGAAACTGTTCGCTCAG	GAGGTTGGTACATCAGAAACACC
58	LAG3	GCAGTGTACTTCACAGAGCTGTC	AAGCCAAAGGCTCCAGTCACCA
59	LAT	ATCCTGGAGCGGCTAAGACTGA	GTTCAGCTCCTGCAGATTCTCG
60	LEFTY2	CCGCGGAAAGAGGTTCAGCCA	GCTGCTCCATGCCGAACACCA
61	LTA	ACACCTTCAGCTGCCCAGACTG	TCCGTGTTTGCTCTCCAGAGCA
62	MAF	AGAAGTTGGTGAGCAGCGGCTT	CACTGATGGCTCCAACTTGCGA
63	MAP2K7	GACCTGGATGTGGTGCTGAAGA	TCTTGAGCTTCTCAGCGCAGGT
64	MAPK8	GACGCCTTATGTAGTGACTCGC	TCCTGGAAAGAGGATTTTGTGGC
65	NFAT5	CCTAATGCCCTGATGACTCCAC	GTTTGCTGAGTTGATCCAACAGAC
66	NFATC1	CACCAAAGTCCTGGAGATCCCA	TTCTTCCTCCCGATGTCCGTCT
67	NFATC2	GATAGTGGGCAACACCAAAGTCC	TCTCGCCTTTCCGCAGCTCAAT
68	NFATC3	AGACAGTCGCTACTGCAAGCCA	GCGGAGTTTCAAAATACCTGCAC
69	NFATC4	GCACCGTATCACAGGCAAGATG	TCAGGATTCCCGCGCAGTCAAT
70	PCGF2	CACTATCGTGGAGTGCCTGCAT	GGTTTTATGGACCTGCACGTCAC
71	PTPRC	CTTCAGTGGTCCCATTGTGGTG	CCACTTTGTTCTCGGCTTCCAG
72	SFTPD	GGACAAAGGCATTCCTGGAGAC	TGAGAGAAAGCAGCCTGGAGGT
73	SOCS1	TTCGCCCTTAGCGTGAAGATGG	TAGTGCTCCAGCAGCTCGAAGA
74	SOCS2	GGTCGGCGGAGGAGCCATCC	GAAAGTTCCTTCTGGTGCCTCTT
75	SPP1	CGAGGTGATAGTGTGGTTTATGG	GCACCATTCAACTCCTCGCTTTC
76	STAT1	ATGGCAGTCTGGCGGCTGAATT	CCAAACCAGGCTGGCACAATTG
77	STAT4	CAGTGAAAGCCATCTCGGAGGA	TGTAGTCTCGCAGGATGTCAGC
78	STAT6	CCTTGGAGAACAGCATTCCTGG	GCACTTCTCCTCTGTGACAGAC
79	TBX21	ATTGCCGTGACTGCCTACCAGA	GGAATTGACAGTTGGGTCCAGG
80	TGFB1	TACCTGAACCCGTGTTGCTCTC	GTTGCTGAGGTATCGCCAGGAA
81	TGFB2	AAGAAGCGTGCTTTGGATGCGG	ATGCTCCAGCACAGAAGTTGGC
82	TGFB3	CTAAGCGGAATGAGCAGAGGATC	TCTCAACAGCCACTCACGCACA
83	TLR4	CCCTGAGGCATTTAGGCAGCTA	AGGTAGAGAGGTGGCTTAGGCT
84	TLR6	ACTGACCTTCCTGGATGTGGCA	TGACCTCATCTTCTGGCAGCTC
85	TMED1	CTTTGACAGCCTCCAGGATGAC	TAGCGTGAGCATCTGGATGCTG
86	TNF	CTCTTCTGCCTGCTGCACTTTG	ATGGGCTACAGGCTTGTCACTC
87	TNFRSF8	ATCTGTGCCACATCAGCCACCA	AAGGTGGTGTCCTTCTCAGCCA
88	TNFRSF9	TCTTCCTCACGCTCCGTTTCTC	TGGAAATCGGCAGCTACAGCCA
89	TNFSF4	CCTACATCTGCCTGCACTTCTC	TGATGACTGAGTTGTTCTGCACC
90	TYK2	GGTTGACCAGAAGGAGATCACC	TCCTCGTCATCCATCTTGCCCT
91	YY1	GGAGGAATACCTGGCATTGACC	CCCTGAACATCTTTGTGCAGCC
92	FoxP3	GGCACAATGTCTCCTCCAGAGA	CAGATGAAGCCTTGGTCAGTGC
93	ACTB	CACCATTGGCAATGAGCGGTTC	AGGTCTTTGCGGATGTCCACGT
94	GAPDH	GTCTCCTCTGACTTCAACAGCG	ACCACCCTGTTGCTGTAGCCAA
95	HPRT1	CATTATGCTGAGGATTTGGAAAGG	CTTGAGCACACAGAGGGCTACA

Table S2, Related to Figure 4. Primer sequences used in single-cell PCR analysis. Gene sequences for human Th1, Th2, and Th3 immune responses (92 sequences) and 3 housekeeping gene sequences were provided by Origene Technologies Inc**.**

Supplemental Experimental Procedures

Generation of humanized mice. NOD.Cg-*Prkdc*^{scid}*Il2rg*^{tm1Wjl} (NSG) mice were obtained from research colonies established by Leonard Shultz at The Jackson Laboratory. Humanized *hSRC and hBLT* mice (12 weeks old) were obtained from the University of Massachusetts (Drs. Dale Greiner and Michael Brehm, University of Massachusetts Medical School). Briefly, humanized *hSRC* mice were developed by injection of T-cell-depleted, umbilical cord blood (UCB) cells containing $5x10^4$ human $CD34^+$ cells via the intracardiac route into 1-3 day-old NSG mice that had received 100 cGy γ-irradiation (Brehm et al., 2010). T-cells were depleted using commercially available anti-human CD3 magnetic bead kits according to the manufacturer's instructions (Miltenyi Biotec, Auburn, CA or Stem Cell Technologies, Vancouver, BC, Canada). Efficiency of T-cell depletion and percentages of $CD34^+$ cells were evaluated by flow cytometry prior to injection into recipient mice and in all experiments revealed less than 0.5% contaminating $CD3^+$ cells in the UCB preparation. *hBLT* mice (12 weeks old) were developed using a method previously described (Covassin et al., 2013). Briefly, male NSG mice (6-8 weeks old) received 200 cGy of γ-irradiation and were then anesthetized and surgically implanted with human fetal liver and thymus under the kidney capsule (McCune et al., 1988). The fetal tissues were phenotyped before implantation by flow cytometry using an antibody specific for HLA-A2 (BB7.2, BD Biosciences) and found to be HLA-A2 negative. Four hours following irradiation, recipient mice were injected intravenously with human fetal liver-derived $CD34^+$ HSCs ($1x10^5$), autologous to the implanted fetal tissue. The fetal liver cell population was depleted of T-cells using commercially available anti-human CD3 magnetic bead kits according to the manufacturer's instructions (Miltenyi Biotec, Auburn, CA or Stem Cell Technologies, Vancouver, BC, Canada), and $CD34^+$ cells were quantified by flow cytometry using an antibody specific for human CD34 (clone 581) (Aryee et al., 2014). All mice were screened for human cell chimerism levels at 12 weeks post-engraftment using antibodies specific for human CD45 (30-F11, BD Biosciences), CD3 (UCHT1, BD Biosciences), and CD20 (2H7, BD Biosciences).

Generation of allogenised mice. For generating *BLT* allogenised mice, female NSG mice at 3-5 days of age were myeloablated with 100 cGy one day prior to the surgical implantation and attachment of allogeneic mouse liver and thymus (<2 mm) to the omentum. This site was chosen due to frailty and small size of the renal capsule in these pups. In addition, lineage-depleted mouse bone marrow cells were injected intra-hepatically. Liver, thymus, and bone marrow originated from young adult female C57BL/6J mice (6 weeks-old). Control mice, FVB/NJ and C57BL/6J,

were purchased from The Jackson Laboratory at 6-8 weeks of age and aged to match our humanized and allogenised mice. All experiments were approved by the Stanford University Administrative Panel of Laboratory Animal Care (APLAC), The Jackson Laboratory and the University of Massachusetts Medical School University Institutional Animal Care and Use Committees (IACUC). All experiments were also approved by Stanford University and University of Massachusetts Medical School Institutional Biosafety Committees.

Labeling of hESCs and miPSCs for bioluminescence imaging. Human ESCs (H9 cell line, HLA-A2^{pos}; obtained from WiCell, Madison, WI) were cultured on Matrigel-coated plates in mTeSR1 medium (Stem Cell Technologies, Vancouver, BC, Canada) as described previously (Ludwig et al., 2006). Mouse iPSCs were created by reprogramming of fibroblasts using a lentiviral based approach for delivery of the four reprogramming factors (*Klf4, Oct4, Sox2 and c-Myc*) (Takahashi and Yamanaka, 2006). Both cell lines were stably transduced with a reporter construct containing the ubiquitin promoter driving firefly luciferase and EGFP as previously described (Pearl et al., 2011).

Differentiation and characterization of hESC-derived endothelial cells (hESC-ECs). Transgenic hESCs were differentiated into ECs by culturing EBs with culture media containing DMEM (Invitrogen) containing 20% knockout serum (Invitrogen), 4.5 g/L L-glutamine (Invitrogen), 1% nonessential amino acids (Invitrogen), 0.1 mM 2-mercaptoethanol (Invitrogen), 50 units/ml penicillin (Invitrogen), 50 μg/ml streptomycin (Invitrogen), 10 ng/ml Activin A (R&D Systems), and 20 ng/ml bone morphogenetic protein-4 (BMP-4) (R&D Systems). The differentiation medium was supplemented with 25 ng/mL vascular endothelial growth factor (VEGF) (R&D system) and 8 ng/mL bFGF (R&D system). At day 4, the EBs were then seeded onto Matrigel-coated dishes and cultured for another 10 days in differentiation media without BMP-4. On day 14, plated EBs were digested and sorted by fluorescence activated cell sorting (FACS) for CD31-positive cells. The CD31^{+} cells were collected and seeded into a six-well plate, coated with 0.2% gelatin, and cultured in EGM-2 medium (Lonza). Characterization of the hESC-ECs was done by immunofluorescence staining for the EC markers CD31, CD144, and laminin and their ability to take up ac-Dil-LDL and form tubes on Matrigel (Huber et al., 2013).

Induction of MHC-I upregulation in hESCs by IFN-γ stimulation or differentiation. For in vitro stimulation of hESCs with IFN-γ, 25 ng/ml of the cytokine was added to the growth media for 48 hr. After 48 hr, hESCs were

harvested and labelled with antibodies for MHC-I (BD Pharmingen, Cat. nr: 555553), SSEA4 (R&D systems, Cat. nr: MAB1435), B2-m (BD pharmingen, Cat. nr: 551337), MHC-II (BD Pharmingen, Cat. nr: 555557), ICAM-1 (BD Pharmingen, Cat. nr: 555511), LFA-3 (BD Pharmingen, Cat. nr: 555921), B7-1 (eBiosciences, Cat. nr: 11-0809), B7-2 (eBiosciences, Cat. nr: 12-0869), B7-H1 (eBiosciences, Cat. nr: 12-5983), and CD40 (eBiosciences, Cat. nr: 11-0409) and analyzed using FACS. To measure MHC-I and other co-stimulatory molecules during differentiation, EBs were harvested at day 14 of differentiation and digested to a single-cell suspension. Cells isolated from these EBs were then stained with the same antibodies as for the IFN-γ stimulation assay and analyzed using FACS.

Cell graft implantation. Mice were placed in an induction chamber and anesthetized using 1-2% isoflurane (Isothesia, Butler Schein) and 98-99% oxygen at a delivery rate of 2 l/min. Cells were mixed with Matrigel High Concentration (BD biosciences) and PBS on a 1:2 ratio and injected s.c. in the lower dorsa, i.m. in the hindlimb, or i.s. in the spleen.

Graft explantation and cell isolation. Grafts were explanted, minced and digested with Liberase (27 WU ml^{-1}; Roche, Indianapolis, IN, USA) and DNase I (0.1%; Roche) in Dulbecco's modified Eagle medium (DMEM) media at 37 °C for 30 min with slight agitation. Digested grafts were passed through a 100-μm strainer and centrifuged at 300*g* for 10 min at 4°C. Cells were counted and prepared for flow cytometry.

Flow cytometric analysis of cytokine production. Cells were isolated from explanted grafts as described above, as well as from murine and human whole blood. Mixed lymphocyte reactions were performed using the Human Intracellular Cytokine Staining Starter Kit (BD Pharmingen). Briefly, the reactions were performed in a 96 well plate, with 5 x 10^5 cells used per reaction. Cells were incubated in one of the following conditions: PMA/ionomycin (10 μL per well), HUVEC lysate (25 μg/well), or hESC-EC lysate (25 μg/well) for 4 hours. The cells were then fixed, permeabilized, and stained with PE-conjugated antibodies for IL-2, IFN-γ, and TNF. Flow cytometry was performed using a FACSAria II SORP flow cytometer (BD Biosciences), and analysis was performed using FlowJo software.

Single-cell PCR analysis. Gene expression of single cells was done using a 96.96 Dynamic Array chip (Fluidigm). Single cells were sorted into each well of a 96-well PCR plate containing 5 ul/well of CellsDirect Reaction Mix buffer (Life Technologies) and 0.2 ul/well of Superase-In RNase inhibitor (Life Technologies) by FACS using murine and

human CD45 markers, isolating human from mouse lymphocytes, and further discrimination was made using human CD3, CD4, CD8, CD45RA, and CD45RO markers (1:100; BD Bioscience or eBioscience). After cell sorting, the PCR plate was briefly centrifuged. To the sorted cells, 0.2 μl/well of SuperScript-III RT/Platinum Taq (Life Technologies), 2.3 μl/well TE Buffer (Life Technologies), and 2.5 μl/well of a mix containing the 95 EvaGreen Assays were added. The 96-well PCR plate was placed in a thermocycler to reverse transcribe mRNA into cDNA (50 °C for 15 min, 70 °C for 2 min) followed by a pre-amplification for 18 cycles (95 °C for 15 s, 60 °C for 4 min). The amplified cDNA was diluted 1:2 with TE Buffer (Life Technologies). 3.38 μl of amplified cDNA were individually mixed with 3.75 μl of TaqMan Universal PCR Master Mix (Life Technologies) and 0.375 μl of 20x Sample Loading Reagent (Fluidigm) and 5 ml from each mix were pipetted into the chip inlets for the samples. 3.75 μl of each TaqMan assay were individually mixed with 3.75 μl of 2x Assay Loading Reagent (Fluidigm) and 5 ml from each mix were pipetted into the chip inlets for the assays. NanoFlex IFC Controler (Fluidigm) was used to load the samples and probes into the M96 chip. The chip was then transferred to a BioMark HD System (Fluidigm) to read the real-time PCR results using the Default-10-min-Hot-start program (50 °C for 2 min, 95 °C for 10 min, and 40 cycles of 95 °C for 15 sec, 60 °C for 1 min). Results were analyzed using the Fluidigm Real-time PCR analysis software.

Immunohistochemistry of explanted grafts. Gastrocnemius muscle containing hESC-EC grafts were explanted, fixed overnight in 4% paraformaldehyde, and transferred to 70% ethanol for 24 hr. Fixed samples were embedded in paraffin and 5 μm sections were cut and stained with hematoxylin and eosin (H&E) for histological analysis. In addition, 10 μm sections were prepared for immunohistochemistry using heat induced epitope retrieval. In brief, paraffin embedded gastrocnemius slides were deparaffinized using 2 washes of 5 min each in Histoclear II (EMS Diasum, PA, USA) followed by an ethanol rehydration series of 100%, 90%, 75%, and 50% EtOH for 3 min each. Antigen unmasking was then performed in 10 mM sodium citrate pH 6.0 in a water bath set to 98° C for 20 min. After antigen retrieval, these sections were blocked with mouse on mouse blocking reagent (Vector Laboratories) and stained with anti-human CD3 (1:100; Abcam), CD31 (1:50; Invitrogen), and anti-GFP (1:500; Abcam). For the CD3 and GFP staining, Alexa-594-conjugated secondary antibodies (1:200; Life Technologies) were used, respectively. An anti-mouse IgG (biotin) and subsequent streptavidin Alexa-488 conjugate was used as a secondary antibody against the anti-CD31 (1:500; Life Technologies). After 3 washes with PBS, slides were mounted with DAPI-containing mounting media (Vectashield, Vector labs) and immunofluorescent images were taken by fluorescent microscopy.

Supplemental References

Aryee, K.E., Shultz, L.D., and Brehm, M.A. (2014). Immunodeficient mouse model for human hematopoietic stem cell engraftment and immune system development. **Methods in Molecular Biology** *1185*, 267-278.

Brehm, M.A., Cuthbert, A., Yang, C., Miller, D.M., DiIorio, P., Laning, J., Burzenski, L., Gott, B., Foreman, O., Kavirayani, A., *et al.* (2010). Parameters for establishing humanized mouse models to study human immunity: analysis of human hematopoietic stem cell engraftment in three immunodeficient strains of mice bearing the IL2rgamma(null) mutation. **Clinical Immunology** *135*, 84-98.

Covassin, L., Jangalwe, S., Jouvet, N., Laning, J., Burzenski, L., Shultz, L.D., and Brehm, M.A. (2013). Human immune system development and survival of non-obese diabetic (NOD)-scid IL2rgamma(null) (NSG) mice engrafted with human thymus and autologous haematopoietic stem cells. **Clinical and Experimental Immunology** *174*, 372-388.

Huber, B.C., Ransohoff, J.D., Ransohoff, K.J., Riegler, J., Ebert, A., Kodo, K., Gong, Y., Sanchez-Freire, V., Dey, D., Kooreman, N.G., *et al.* (2013). Costimulation-adhesion blockade is superior to cyclosporine A and prednisone immunosuppressive therapy for preventing rejection of differentiated human embryonic stem cells following transplantation. **Stem Cells** *31*, 2354-2363.

Ludwig, T.E., Levenstein, M.E., Jones, J.M., Berggren, W.T., Mitchen, E.R., Frane, J.L., Crandall, L.J., Daigh, C.A., Conard, K.R., Piekarczyk, M.S., *et al.* (2006). Derivation of human embryonic stem cells in defined conditions. **Nature Biotechnol** *24*, 185-187.

McCune, J.M., Namikawa, R., Kaneshima, H., Shultz, L.D., Lieberman, M., and Weissman, I.L. (1988). The SCID-hu mouse: murine model for the analysis of human hematolymphoid differentiation and function. **Science** *241*, 1632-1639.

Pearl, J.I., Lee, A.S., Leveson-Gower, D.B., Sun, N., Ghosh, Z., Lan, F., Ransohoff, J., Negrin, R.S., Davis, M.M., and Wu, J.C. (2011). Short-term immunosuppression promotes engraftment of embryonic and induced pluripotent stem cells. **Cell Stem Cell** *8*, 309-317.

Takahashi, K., and Yamanaka, S. (2006). Induction of pluripotent stem cells from mouse embryonic and adult fibroblast cultures by defined factors. **Cell** *126*, 663-676.
